# Multi-Omics Analysis of Drought and Growth Responses in *Nitraria sibirica*: Insights from Habitat-Specific and Developmental Comparisons

**DOI:** 10.3390/plants15162422

**Published:** 2026-08-08

**Authors:** Yaling Chang, Fu Ran, Qingshan Fan, Fang Yang, Xiaoming Bai, Guanghui Lv, Ting Wang

**Affiliations:** 1 College of Grassland Science, Gansu Agricultural University, Lanzhou 730070, China; changyl@gsau.edu.cn (Y.C.); ranf@gsau.edu.cn (F.R.); fanqs@gsau.edu.cn (Q.F.); wangting921221@163.com (T.W.); 2 School of Ecology and Environment, Xinjiang University, Urumqi 830017, China; yf13579863579@163.com

**Keywords:** *Nitraria sibirica*, shrub, drought stress, genes, metabolites, growth

## Abstract

Global climate change-driven drought aggravation seriously threatens the survival and distribution of desert shrubs. *Nitraria sibirica* Pall. has outstanding drought tolerance, yet its growth adaptation strategies and internal regulatory mechanisms under different soil moisture conditions lack systematic research. This study combined physiological determination with transcriptome and metabolome analyses to investigate its physiological responses and molecular regulation mechanisms at different growth stages and habitats. *Nitraria sibirica* from riparian habitats exhibited lower leaf organic carbon and higher total nitrogen. Desert-grown *N. sibirica* possessed far more drought-responsive differentially expressed genes and metabolites than riparian ones. Hormone signal transduction and lipid metabolism dominated regulation in riparian habitats, whereas secondary metabolite biosynthesis and amino acid metabolism—especially flavonoid biosynthesis and tryptophan metabolism—were core in desert habitats. Its drought response displayed distinct stage-specificity, with hormone signal transduction most enriched in drought-responsive genes across growth periods. This study clarifies the stress adaptation mechanisms of desert shrubs and provides theoretical support for germplasm evaluation and vegetation restoration in arid regions.

## 1. Introduction

As a key component of desert ecosystems, shrubs play a critical role in controlling wind erosion, conserving soil and water, maintaining regional biodiversity, and stabilizing ecosystem structure and function. However, intensified drought driven by global climate change poses a severe threat to the survival and distribution of desert shrubs. When exposed to environmental stress, plants typically respond via adjustments in morphological structure, physiological metabolism, and molecular regulation, and such adaptive strategies operate throughout their entire growth and development [1]. Therefore, investigating plant responses to drought stress requires consideration of stage-specific responses across different developmental periods. Moreover, under drought-induced resource limitation, plants must make adaptive trade-offs in resource allocation to cope with unfavorable conditions. For instance, under drought stress, plants may reduce investment in vegetative growth and reallocate more resources toward stress defense pathways [2,3].

*Nitraria sibirica* is a dominant shrub species in desert ecosystems, exhibiting strong resistance to drought and wind–sand disturbance. It is widely distributed across arid and semi-arid regions of northwestern China. In its natural habitat, *N. sibirica* can form large sand-fixing mounds through its growth form, enabling it to survive under extreme drought conditions while maintaining critical ecological functions [4]. Therefore, systematic exploration of the drought response mechanisms in *N. sibirica* not only provides theoretical support for screening drought-resistant germplasm resources of desert shrubs, but also offers scientific guidance for the restoration and reconstruction of desert vegetation in arid regions, with substantial theoretical and practical significance.

Single-omics approaches dissect plant stress responses from a single molecular dimension, disrupting the inherent crosstalk between gene transcription and metabolite accumulation and precluding complete elucidation of plant adaptive regulatory networks [5]. By contrast, integrative multi-omics facilitates cross-validation and complementary analysis spanning transcriptional and metabolic tiers, allowing systematic dissection of the molecular mechanisms driving plant stress acclimation [6,7]. To date, combined transcriptomic and metabolomic profiling has been widely applied to resolve molecular cascades controlling plant development and drought responsiveness, establishing itself as a robust and standard methodology for abiotic stress research in plants. *Nitraria sibirica*, a typical xerophyte naturally distributed across arid and semi-arid habitats, has evolved sophisticated strategies to cope with persistent drought. However, the synergistic regulatory cascade linking functional genes and metabolites to its drought adaptation remains largely uncharacterized. To fill this research gap, the present study uses an integrative multi-omics strategy that integrates habitat variation and developmental progression as key experimental variables to unravel the core molecular regulatory landscape underlying drought tolerance in *N. sibirica*. This analytical framework overcomes the inherent limitations of single-omics techniques and provides a robust theoretical basis for holistic exploration of the evolutionary mechanisms responsible for its drought-adaptive traits.

Previous studies have demonstrated that *N. sibirica* can adapt to long-term soil water deficit through physiological strategies, including reduced photosynthetic rate, enhanced antioxidant system activity, and accumulation of osmotic adjustment substances [8]. In addition, our team has previously analyzed the metabolomic responses of *N. sibirica* to drought stress [9]. Nevertheless, current research remains limited regarding the integrated transcriptional–metabolic regulatory networks underlying growth, development, and drought adaptation in *N. sibirica*. Accordingly, this study was conducted on the northern bank of the Aqikesu River in the Abihu Lake Basin, using *N. sibirica* populations distributed along a natural soil water gradient as the study material. By combining leaf stoichiometric analyses with transcriptomic and metabolomic sequencing, we compared physiological, gene, and metabolic responses of *N. sibirica* across two contrasting habitat types (moisture gradients) and three developmental stages (vegetative, flowering, and fruiting). This study aims to clarify ecological adaptation strategies of plants in arid regions from a multi-omics perspective, thereby providing a theoretical foundation for the conservation and restoration of desert vegetation. The objectives were to reveal: (1) stoichiometric adaptation strategies in *N. sibirica* leaves under drought stress; (2) gene and metabolite expression patterns during the dynamic growth of *N. sibirica* in the two water habitats; (3) coordinated regulatory mechanisms underlying drought responses and their stage-specific characteristics at the transcriptomic and metabolomic levels.

## 2. Results

### 2.1. Chemical Stoichiometric Adaptation Characteristics of the Leaves of N. sibirica

**Figure 1:**
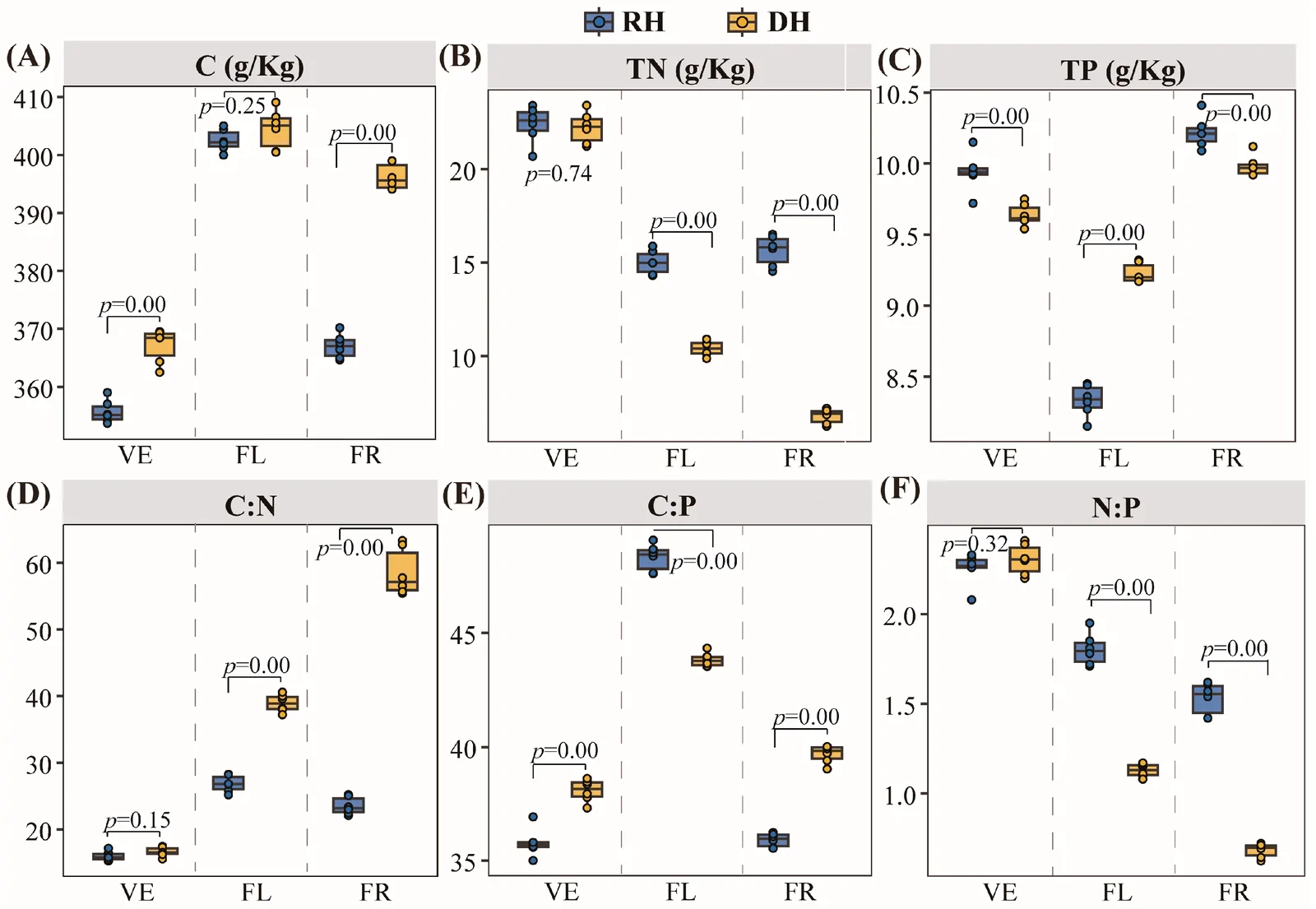
Chemical composition characteristics of the leaves of *N. sibirica*. (**A**) Organic carbon (**C**); (**B**) total nitrogen (TN); (**C**) total phosphorus (TP); (**D**) carbon–nitrogen ratio (C:N); (**E**) carbon–phosphorus ratio (C:P); (**F**) nitrogen–phosphorus ratio (N:P). VE, FL, and FR represent the vegetative, flowering, and fruiting stages, respectively. RH and DH represent the riparian habitat and desert habitat, respectively. The circles represent the original data values of each indicator for individual samples.

Compared with the desert habitat, the organic carbon (C) and carbon–nitrogen (C:N) ratio contents of *N. sibirica* leaves were lower in the riparian habitat, while the contents of total nitrogen (TN), carbon–phosphorus (C:P) ratio, and nitrogen–phosphorus (N:P) ratio (excluding the vegetative period) were significantly higher (Figure 1, Table S1). Furthermore, as plants developed, leaf C content displayed a trend of initial increase followed by decrease, ranging from 353.70 to 409.08 g/kg (Figure 1A), whereas total phosphorus (TP) content showed the opposite pattern, varying from 8.15 to 10.41 g/kg (Figure 1C). In contrast to C and TP, TN content peaked at the VE (Figure 1B). Two-way ANOVA revealed that the habitat × developmental stage interaction exerted no significant effect on leaf stoichiometric traits (except for the C:P), although both main effects were significant. Habitat alone showed no significant influence on TP content or the C:P ratio (Table S1).

Correlation analysis among various chemical composition indices demonstrated that C exhibited a significant negative correlation with TN and TP, whereas no significant correlation was observed between TN and TP (Table S2). The C:P ratio was significantly correlated with both C and TN concentrations, with a stronger correlation with TN than with C. The N:P ratio showed a stronger association with TN, while the C:P ratio was more strongly correlated with TP than with C.

### 2.2. Overview of Transcription and Metabolism Sequencing Data

**Figure 2:**
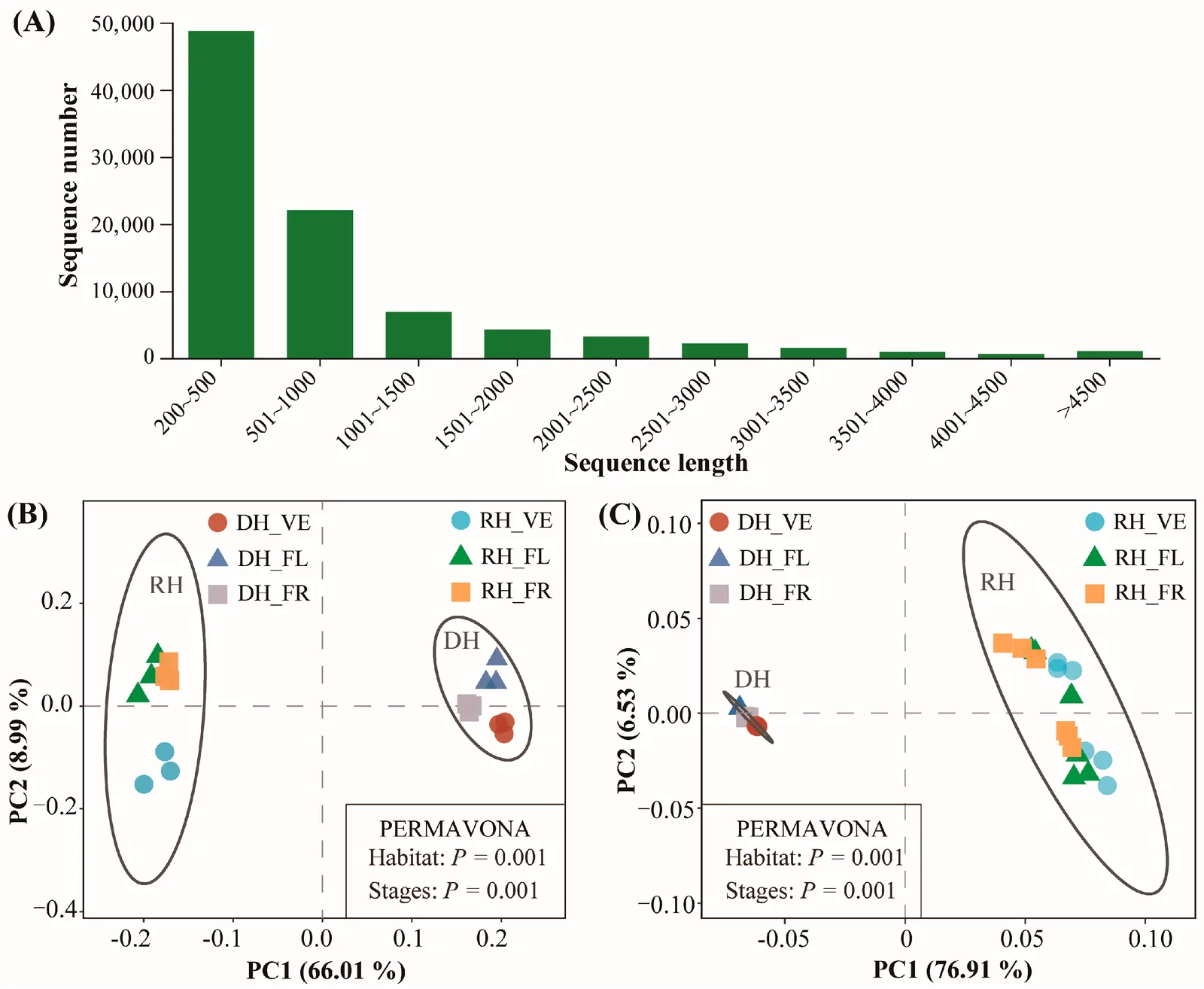
Transcript length distribution (**A**) and expression profile analysis of transcriptomic (**B**) and metabolomic (**C**) samples. RH and DH represent riparian habitat and desert habitat, respectively; VE, FL, and FR represent vegetative, flowering and fruiting, respectively. PERMAVONA, permutational multivariate analysis of variance. Subfigures (**A**,**B**) reproduced from Chang, Y.L., Lv, G.H. Plant Physiol. Biochem. 2024, 206, 108265 [8]. Copyright © 2024 Elsevier Masson SAS. All rights reserved.

The RNA-seq data and corresponding analytical graphs used in this study were obtained from the same batch of samples as reported in our previous work [8]. Transcriptomic sequencing of *N. sibirica* leaves revealed that the GC content of each sample ranged from 44.69% to 46.06%, and the Q30 base percentage exceeded 92.50% (Table S3). All clean reads were de novo assembled using Trinity software. The assembled unigenes exhibited a length distribution of 200~500 bp, which accounted for the highest sequence abundance (Figure 2A). The quality of the transcriptomic data fully satisfied the criteria for subsequent bioinformatic analyses. To validate the RNA-Seq results, qRT-PCR analysis was conducted on 10 DEGs. The qRT-PCR data was highly consistent with the RNA-Seq data and showed a significant positive correlation (R^2^ = 0.86), thereby enhancing the reliability of the RNA-Seq data (Figure S1). The qRT-PCR validation data of the tested genes were published in our prior study, with identical plant materials, samples and experimental treatments used across both studies [8].

Principal component analysis of transcriptomic and metabolomic profiles revealed that sample separation was primarily driven by habitat divergence and was also influenced by developmental stage (Figure 2B,C). Samples from the two habitats were clearly distinguished along the first principal component axis, with gene and metabolite profiles exhibiting less variation in the desert habitat than in the riparian habitat (Figure 2B,C). Furthermore, the cumulative contribution rate of the first two principal components exceeded 75%. Multivariate analysis of variance (MANOVA) confirmed significant differences in metabolic profiles across habitats and developmental stages (*p* = 0.001) (Figure 2B,C).

### 2.3. Transcriptomic Responses to Growth Stages and Drought

**Figure 3:**
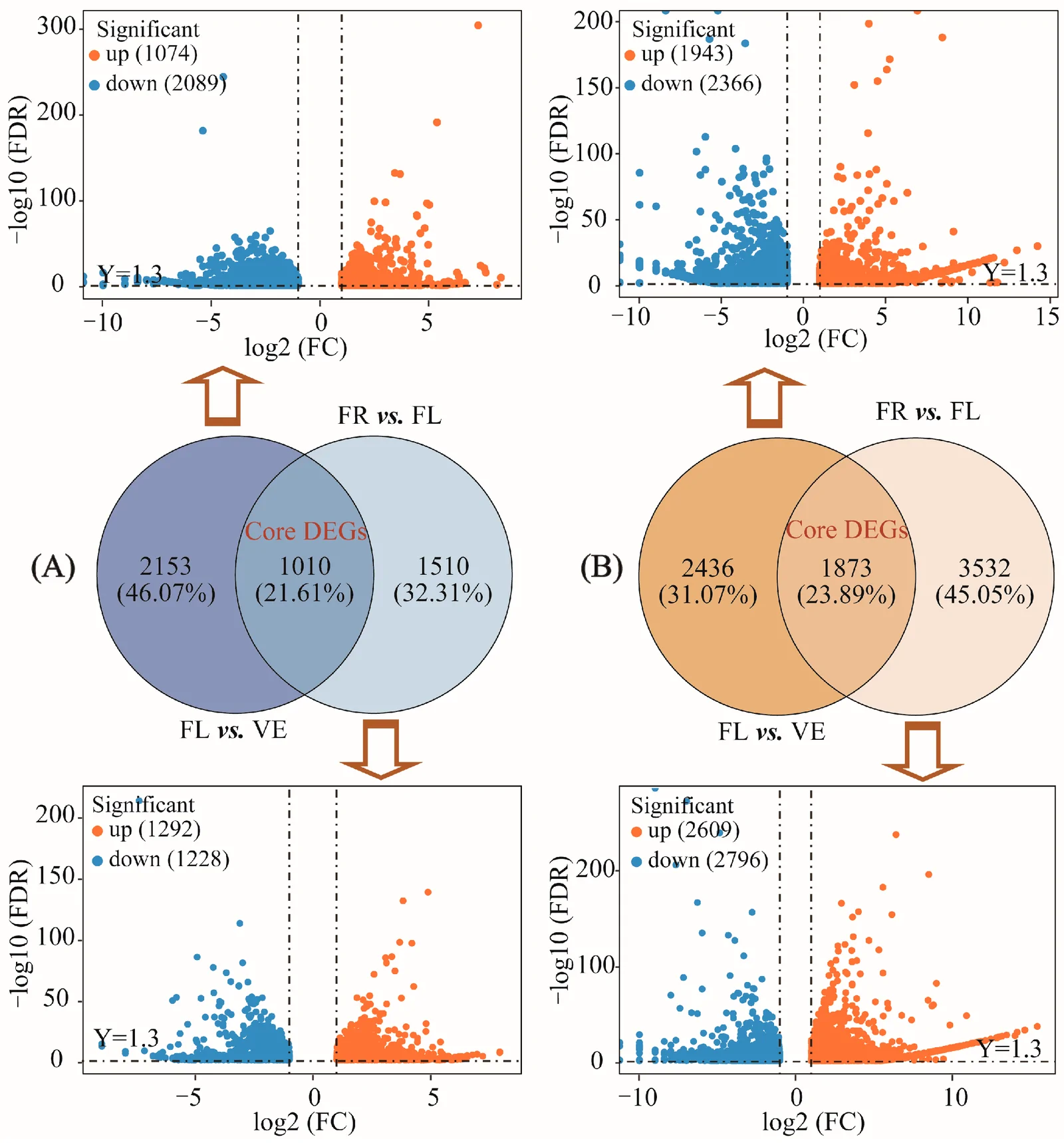
Venn diagram and volcano plot of growth response DEGs. (**A**) Riparian habitat; (**B**) desert habitat. VE, FL, and FR represent vegetative, flowering and fruiting, respectively. Different colors in the Venn diagram represent the DEGs of different comparison groups. The overlapped portion represents the number of genes shared by the two gene sets, while the un-overlapped portion represents the number of genes unique to the gene set. Each dot in the volcano map represents a specific gene, with the dot on the left representing a gene with downregulated expression and the dot on the right representing a gene with upregulated expression.

Analysis of DEGs associated with growth responses in *N. sibirica* revealed that with the progression of leaf developmental stages, the number of DEGs gradually decreased in the riparian habitat comparison groups (3163 DEGs in FL vs. VE, 2520 DEGs in FR vs. FL), whereas it gradually increased in the desert habitat groups (4309 DEGs in FL vs. VE, 5405 DEGs in FR vs. FL) (Figure 3). Overall, more growth-related DEGs were identified in the desert habitat than in the riparian habitat, suggesting that *N. sibirica* induces a greater number of DEGs to adapt to arid environments. Moreover, DEG expression patterns were regulated by developmental stage: in both habitats, DEGs in the FL vs. VE comparison were predominantly downregulated, whereas in the FR vs. FL comparison, DEGs were mainly upregulated in the riparian habitat but primarily downregulated in the desert habitat (Figure 3).

Analysis of drought-responsive DEGs in *N. sibirica* showed that the number of DEGs was highest at the VE stage, with 21,737 DEGs (14,265 upregulated and 7472 downregulated), and lowest at the FR stage, with 18,402 DEGs (12,742 upregulated and 5660 downregulated) (Figure S2). In addition, 9403 common drought-responsive DEGs were shared across the three stages, while 5725, 4088, and 4846 DEGs were specifically expressed at the VE, FL, and FR stages, respectively (Figure S2). These results indicate that the drought adaptation strategies of *N. sibirica* are stage-dependent.

**Figure 4:**
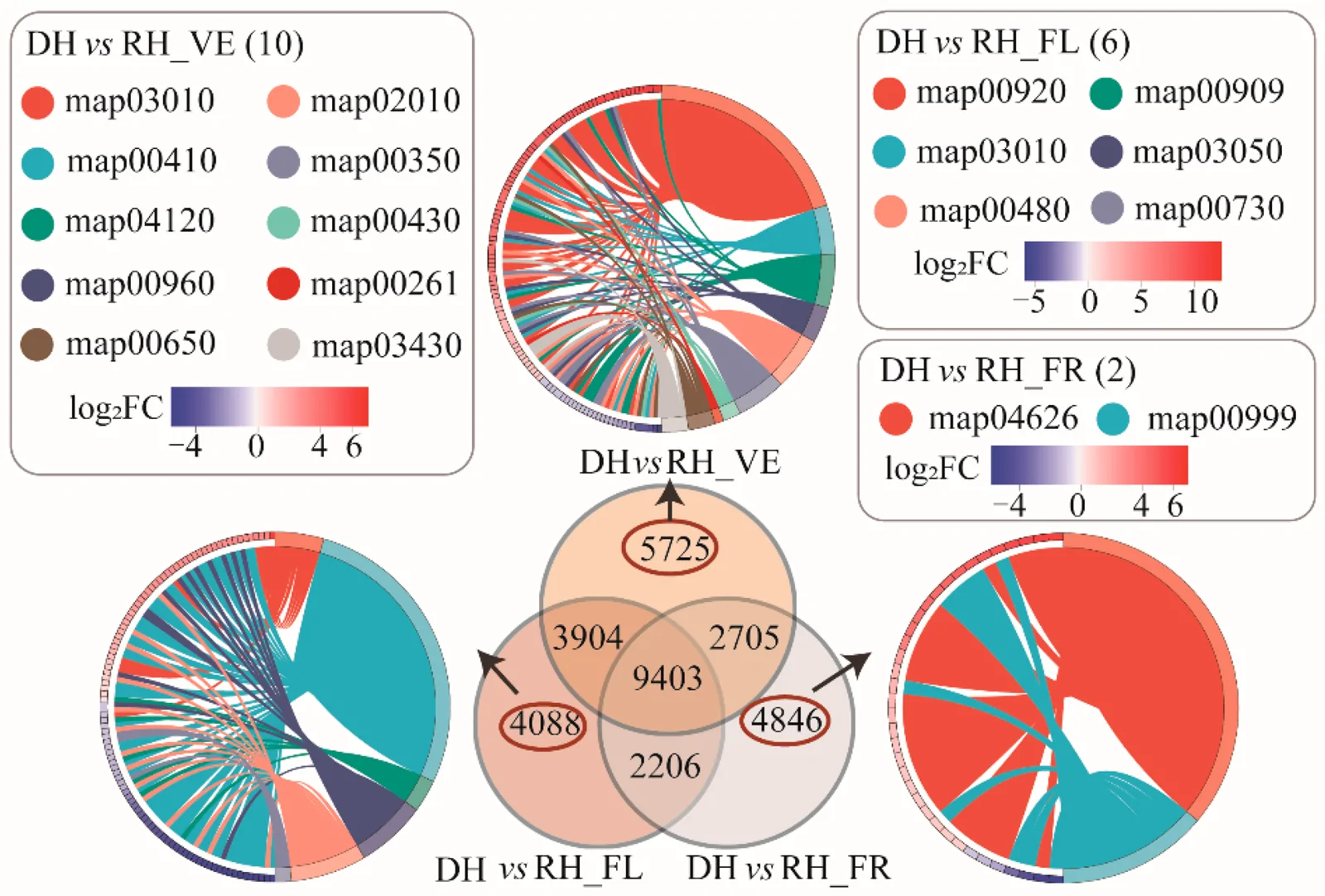
Chord diagram for enrichment analysis of drought-responsive DEGs. RH and DH represent riparian habitat and desert habitat, respectively; VE, FL, and FR represent vegetative, flowering and fruiting, respectively. The outer ring segments represent different functional categories, while the inner ribbons indicate the overlapping and distribution patterns of the DEGs across various functional modules. The Venn diagram shown in this figure is reproduced from Chang, Y.L., Lv, G.H. Plant Physiol. Biochem. 2024, 206, 108265 [8]. Copyright © 2024 Elsevier Masson SAS. All rights reserved.

To further characterize the stage-specificity of drought stress responses, KEGG (Kyoto Encyclopedia of Genes and Genomes) pathway annotation was performed on the stage-specific drought-responsive DEGs (Figure 4). Genes responsive to drought stress were involved in the most diverse pathways at the VE stage, where DEGs were mainly annotated to 10 pathways: genetic information processing (map03010, map03430, and map04120), ABC transporters (map02010), amino acid metabolism (map00410 and map00350), taurine and cysteine metabolism (map00430), pyridine alkaloid biosynthesis (map00960), monocyclic sesquiterpenoid biosynthesis (map00261), and butanoate metabolism (map00650) (Figure 4). DEGs at the FL stage were enriched in sulfur metabolism (map00920), ribosome (map03010), glutathione metabolism (map00480), thiamine metabolism (map00730), sesquiterpenoid and triterpenoid biosynthesis (map00909), and proteasome (map03050) pathways (Figure 4). DEGs at the FR stage were enriched in biosynthesis of other secondary metabolites (map00999) and plant–pathogen interaction (map04626) pathways (Figure 4).

### 2.4. Metabolomic Responses to Growth Stages and Drought

**Figure 5:**
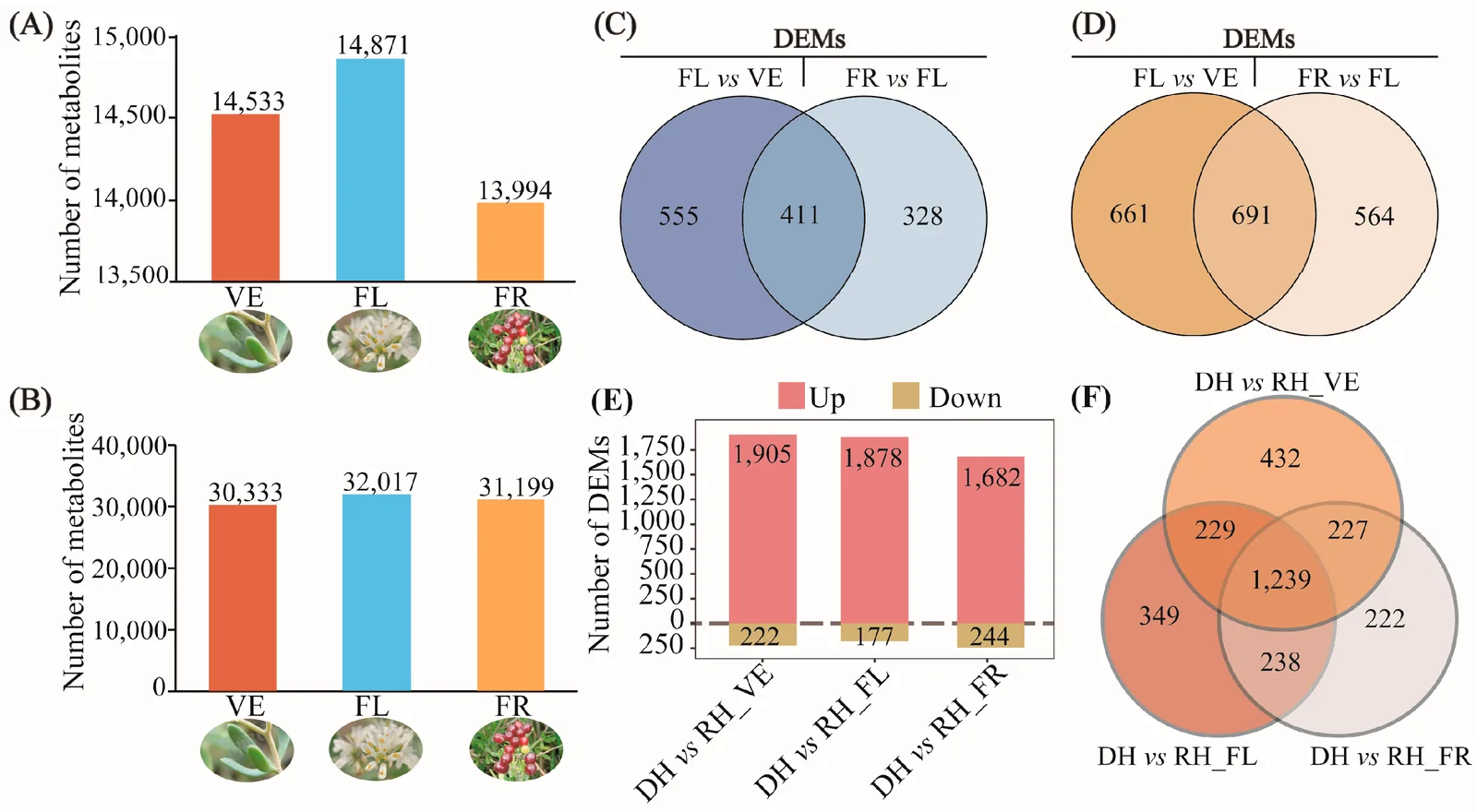
Metabolite identification. Metabolic products in the (**A**) riparian and (**B**) desert habitats; Venn diagram of DAMs numbers of (**C**) riparian and (**D**) desert habitats; drought response DAMs (**E**) upregulated and downregulated shown as a bar chart and a (**F**) Venn diagram. RH and DH represent riparian habitat and desert habitat, respectively; VE, FL, and FR represent vegetative, flowering and fruiting, respectively. Subfigures (**A**,**B**) reproduced from reproduced from Chang, Y.L., Lv, G.H., He, X.M. Environ. Exp. Bot. 2024, 221, 105720 [9]. Copyright © 2024 Elsevier Masson SAS. All rights reserved.

The metabolites in both habitats were most abundant during the FL stage (Figure 5A,B). The identification results of growth response-related DAMs showed that in the mixed positive and negative ion mode, 966 and 739 DAMs were identified in the FL vs. VE and FR vs. FL comparison groups of the riparian habitat, respectively. In contrast, 1352 and 1255 DAMs were detected in the corresponding comparison groups of the desert habitat (Figure 5C,D). Overall, the total number of DAMs in the desert habitat was significantly higher than that in the riparian habitat. During the growth process, the number of up-regulated metabolites in *N. sibirica* gradually decreased, while the number of down-regulated metabolites gradually increased (Figure S3).

The number of up-regulated DAMs in *N. sibirica* was much higher than that of down-regulated DAMs during the three stages of drought stress (Figure 5E). Through pairwise comparisons, a total of 1239 co-expressed responsive DAMs were identified among the three comparison groups, whereas 5725, 4846, and 4088 stage-specific DAMs were detected in the three stages, respectively (Figure 5F). For these 1239 co-expressed DAMs, Human Metabolome Database classification revealed that the most abundant categories were lipids and lipid-like molecules (26.11%) and organic acids and their derivatives (18.78%), with hydrocarbons accounting for the lowest proportion (0.09%) (Figure S4). Additionally, 73 DAMs were not classified into any category (Figure S4).

The KEGG pathway annotations of the three-stage responses to drought-specific DAMs showed that the metabolites involved in the pathways affected by drought stress were most widely distributed in the VE stage. Among them, the DAMs of VE were significantly annotated in linoleic acid (map00591), phenylalanine metabolism (map00360), nucleotide metabolism (map01232), phenylpropanoid biosynthesis (map00940), and glycerophospholipid metabolism (map00564) (Figure S5A); the DAMs of FL were significantly enriched in categories such as glycerophospholipid metabolism (map00564), phosphatidylinositol signaling system (map04070), folic acid synthesis (map00790), tryptophan metabolism (map00380), α-linolenic acid metabolism (map00592), and the biosynthesis of other secondary metabolites (Figure S5B); the DAMs of FR were significantly enriched in categories such as ABC transporters (map02010), acetaldehyde and dicarboxylic acid metabolism (map00630), and amino acid pathways (Figure S5C).

### 2.5. Integrated Analysis of Transcriptome and Metabolome

#### 2.5.1. Correlation Analysis of DEGs and DAMs

**Figure 6:**
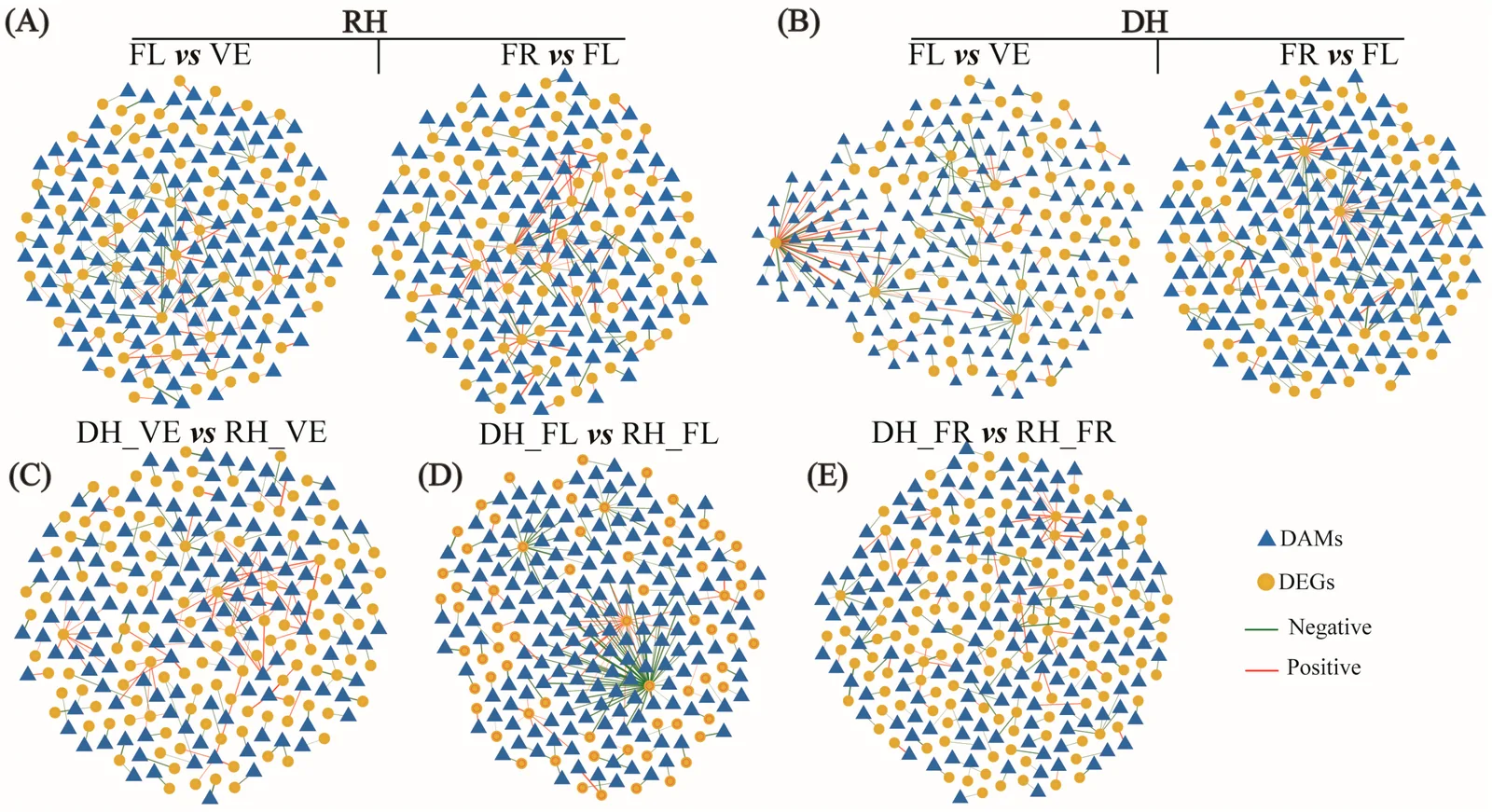
Correlation network analysis of DEGs and DAMs. DEGs and DAMs related to growth response in the (**A**) riparian and (**B**) desert habitats; DEGs and DAMs related to drought response during the (**C**) VE; (**D**) FL and (**E**) FR. RH and DH represent riparian habitat and desert habitat, respectively; VE, FL, and FR represent vegetative, flowering and fruiting, respectively.

Network analysis revealed that growth-responsive DEGs and DAMs mostly exhibited positive correlations in the FL vs. VE comparison of the riparian habitat, whereas in other comparison groups, they primarily showed negative correlations (Figure 6A,B, Table S4). The associated networks of drought-responsive DEGs and DAMs at each stage were mainly characterized by negative correlations (Figure 6C–E, Table S5). The above network topology structure shows that some of DEGs act as core hub nodes, capable of simultaneously targeting multiple DAMs. They are key regulatory factors that mediate transcriptional changes and drive metabolic remodeling. At the same time, there are a large number of DEGs and DAMs that are not connected to each other in the network, indicating that these molecules do not form a coordinated response at the transcriptional and metabolic levels. Their expression or accumulation processes are relatively independent and may be involved in other physiological processes or regulated by multiple-level mechanisms such as post-transcriptional modifications and protein regulation. Additionally, orthogonal partial least-squares comprehensive analysis further confirmed a strong correlation between growth- and drought-responsive DEGs and DAMs (Figure S6).

#### 2.5.2. Analysis of Common Pathways Annotated by Growth Response DEGs and DAMs

The common KEGG functional enrichment analysis of DAMs and DEGs revealed that the growth response of *N. sibirica* in riparian habitats was mainly regulated by hormone signal transduction and lipid metabolism pathways. Specifically, in the FL vs. VE comparison group, DAMs and DEGs were jointly enriched in 6 pathways, including plant hormone signal transduction (map04075), phenylalanine metabolism (map00360), lipid metabolism (map00073, map00592, map00591), and phenylpropanoid biosynthesis (map00940) (Table S6). In contrast, in the FR vs. FL comparison group, they were jointly enriched in 4 pathways, mainly involving plant hormone signal transduction (map04075) and lipid metabolism (map00592, map00073, map00591) (Table S6).

On the other hand, the adaptive growth of *N. sibirica* in desert habitats was dominated by secondary metabolite biosynthesis and amino acid metabolism pathways (Table S6). In the FL vs. VE comparison, DAMs and DEGs were jointly and significantly enriched in 6 pathways, including plant hormone signal transduction (map04075), amino acid metabolism (map00380, map00360, map00330), and secondary metabolite biosynthesis (map00940, map00941) (Table S6). In the FR vs. FL comparison, the 3 jointly enriched pathways were phenylpropanoid biosynthesis (map00940), flavonoid biosynthesis (map00941), and tryptophan metabolism (map00380) (Table S6). In summary, the regulatory pathways for the growth adaptation of *N. sibirica* differ between the two habitats.

In the desert habitat, the unique metabolic pathways associated with the growth response were flavonoid biosynthesis (map00941) and tryptophan metabolism (map00380). In the flavonoid biosynthesis pathway, we observed a continuous decrease in the expression levels of the flavonol synthase (TRINITY_DN4779_c0_g2), licodione synthase-like (TRINITY_DN21071_c0_g1), luteolin, and quercetin (Table S7). In the tryptophan metabolism pathway, the expression levels of (R, S)-reticuline 7-O-methyltransferase-like (TRINITY_DN9396_c0_g1) and 6-Hydroxymelatonin were found to increase (Table S8).

#### 2.5.3. Analysis of Common Pathways Annotated by Drought Response DEGs and DAMs

The KEGG annotation results indicated that the number of significantly enriched pathways shared by drought-responsive DAMs and DEGs gradually decreased with the progression of the developmental process (Table S9). Specifically, there were 10 significantly enriched shared pathways between DAMs and DEGs during the VE stage, 7 pathways during the FL stage, and 6 pathways during the FR stage. Notably, plant hormone signal transduction (map04075), tryptophan metabolism (map00380), alkaloid biosynthesis (map00960), and phenylpropanoid biosynthesis (map00940) played crucial roles in the response of *N. sibirica* to drought stress (Table S9).

**Figure 7:**
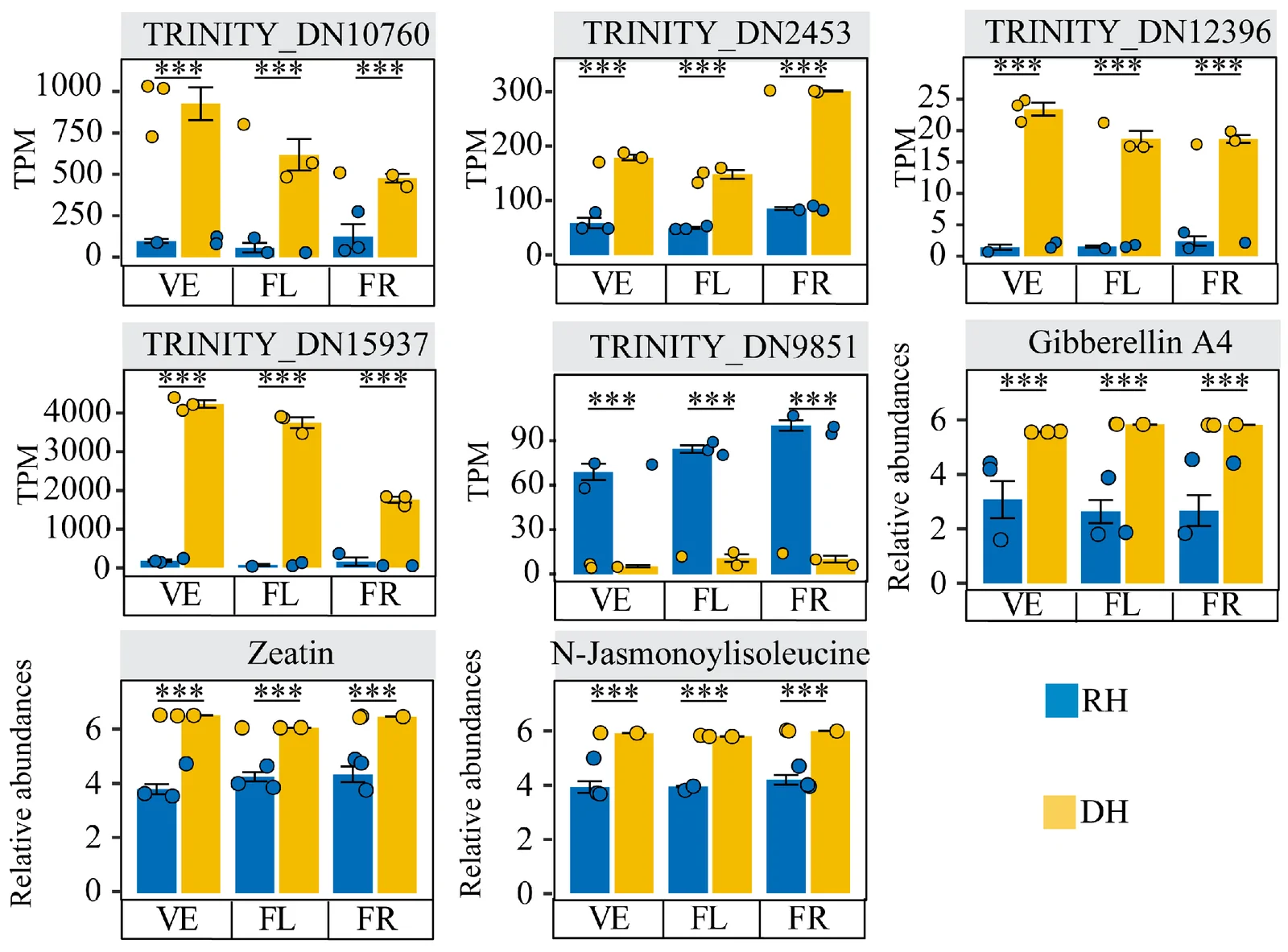
Differentially expressed genes and differential metabolites in hormone signaling pathways. TPM, transcripts per kilobase per million mapped reads. Relative abundances are calculated by normalizing against the original mass spectral peak areas. VE, FL and FR represent the vegetative, flowering and fruiting stages, respectively. Statistical comparisons were performed between two different habitats at an identical developmental stage. *** denotes significant difference at *p* < 0.001 based on F-test. Individual scatter points represent biological replicates; bar height corresponds to mean value, and error bars indicate standard error. TRINITY_DN10760_c0_g1 and GKU86329.1 belong to the AP2 family. TRINITY_DN2453_c0_g1 is the ethylene response transcription factor XP_006420203.1. TRINITY_DN12396_c0_g1 is KAH9698875.1. TRINITY_DN15937_c0_g3 is an auxin response factor KAG5229376.1. TRINITY_DN9851 is a NADH flavin oxidoreductase.

The plant hormone signaling pathway was highly enriched among the most drought-responsive DEGs across all three stages (Table S9). Within this pathway, the expression levels of GKU86329.1 (TRINITY_DN10760_c0_g1) from the AP2 family, the ethylene response transcription factor XP_006420203.1 (TRINITY_DN2453_c0_g1), and KAH9698875.1 (TRINITY_DN12396_c0_g1) were significantly upregulated, whereas the expression level of the auxin response factor KAG5229376.1 (TRINITY_DN15937_c0_g3) was significantly downregulated (Figure 7). Furthermore, the expression levels of three metabolites associated with the plant hormone signaling pathway—including gibberellin A4, zeatin, and N-methyl jasmonoylisoleucine—were significantly elevated in response to drought stress (Figure 7).

**Figure 8:**
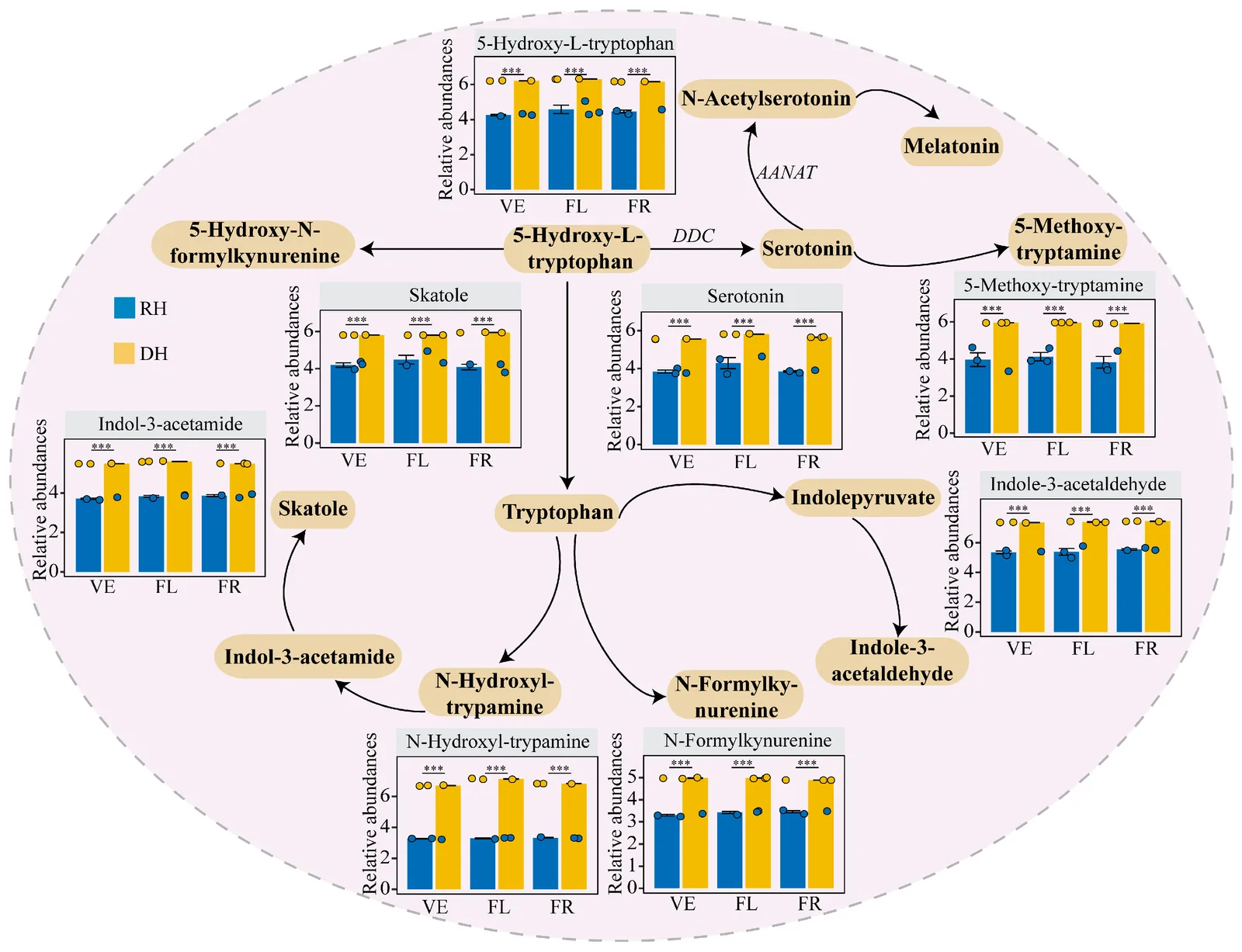
DAMs and DEGs involved in tryptophan metabolic pathways. Relative abundances were calculated by normalization of the original mass spectral peak areas. VE, FL and FR represent the vegetative, flowering and fruiting stages, respectively. Statistical comparisons were performed between the two different habitats for the identical developmental stage. *** denotes significant difference at *p* < 0.001 based on F-test. Individual scatter points represent biological replicates; bar height corresponds to mean value, and error bars indicate standard error. RH and DH represent the riparian habitat and desert habitat, respectively.

In the tryptophan metabolic pathway (Figure 8), the relative abundances of 5-Hydroxy-L-tryptophan, skatole, serotonin, 5-methoxytryptamine, indole-3-acetamide, indole-3-acetaldehyde, N-hydroxyl-trypamine, and N-formylkynurine significantly increased under drought stress (*p* < 0.001). Additionally, drought stress significantly increased the expression levels of methyltransferases (TRINITY_DN14664_c0_g1, TRINITY_DN6011_c1_g2, and TRINITY_DN15389_c0_g1), acetyltransferase (TRINITY_DN9072_c0_g1), and aldehyde dehydrogenase (TRINITY_DN8157_c0_g1), while significantly inhibiting the expression of ACAT1L protein (TRINITY_DN8900_c0_g1) (Table S10).

Drought stress significantly affected the expression levels of DAMs and DEGs involved in the alkaloid synthesis pathway (Figure S7A,B). Among the DAMs, the expression levels of tropineone, allysine, and pipecolic acid were significantly decreased, while those of scopolamine, swainsonine, sparteine, L-isoleucine, and methylisopelletierine were significantly increased (*p* < 0.001). For the DEGs involved in the alkaloid synthesis pathway, most genes related to tropineone reductase and tyrosine aminotransferase were upregulated; however, the expression levels of some tropineone reductase-related genes (TRINITY_DN2851_c0_g2 and TRINITY_DN4241_c0_g1) and tyrosine aminotransferase-related genes (TRINITY_DN1716_c0_g2 and TRINITY_DN7706_c0_g2) were inhibited. Notably, the uncharacterized protein TRINITY_DN15973_c0_g1 exhibited stage-specific expression: its expression was downregulated during the VE stage under drought stress but upregulated during the FL and FR stages.

Under drought stress, 5 DAMs and 46 DEGs were involved in the phenylpropanoid biosynthesis pathway (Figure S7C,D). Among the metabolites, the expression level of cinnamaldehyde was decreased, while those of coniferaldehyde, 5-hydroxyferulic acid, coniferin, and coniferyl acetate were increased (Figure S7D). Additionally, drought stress led to the upregulation of phenylalanine ammonia-lyase (PAL, TRINITY_DN11009_c0_g1), cytochrome P450 (TRINITY_DN14310_c0_g1), and O-methyltransferase (TRINITY_DN20567_c0_g1) (Table S11).

## 3. Discussion

### 3.1. Variations in Nutritional Elements of N. sibirica Leaves on Growth and Drought Adaptation

The accumulation and distribution of nutrient elements in leaves are direct manifestations of a plant’s environmental adaptation strategies. Dynamic changes in chemical composition characteristics are jointly determined by plant physiological metabolism and environmental regulation. Analyzing this mechanism is of great significance for revealing the adaptation mechanism of *N. sibirica* and optimizing vegetation management. The influence of habitat types on the chemical composition characteristics of *N. sibirica* leaves mainly stems from differences in water and nutrient supply between riparian and desert habitats. The riparian habitat has abundant water and rich organic matter and nutrients in the soil, which is conducive to the plant absorbing more nitrogen. Therefore, the total nitrogen content in the leaves of *N. sibirica* is relatively high (Figure 1B). In contrast, the desert habitat is usually limited by water and nutrient supply, and plants need to utilize limited resources more efficiently for growth. Thus, plants in the desert habitat may accumulate more organic carbon to cope with stress conditions such as drought (Figure 1A). Nutrient elements play an important role in plant growth and various physiological regulatory functions, and their variations can reflect adaptive strategies in response to environmental conditions and changes in species composition [10]. Plants mainly respond to environmental changes through endogenous physiological regulation, nutrient allocation remodeling, and metabolic pathway reprogramming. The nutrient homeostasis of leaves is the foundation for maintaining normal plant life activities. Changes in the environment cause variations in the carbon and nitrogen contents of leaves, which may disrupt the original carbon and nitrogen metabolic balance within cells, thereby activating upstream signaling pathways, inducing differential expression of a large number of stress-related genes, and promoting global changes in primary and secondary metabolic pathways.

### 3.2. Differences in Metabolic Pathways of Growth Response Genes and Metabolites of N. sibirica in the Two Habitats

Drought conditions pose a survival challenge for plants, and plants adapt and resist this stress by adjusting their gene expression patterns, activating or inhibiting the expression of specific genes. This regulation can enhance the plant’s adaptability to the drought environment. Some genes may be activated to enhance the plant’s drought resistance, such as those involved in the synthesis of dehydration protection substances, regulating root growth and water utilization efficiency [11]. In this study, the specific enriched pathways in the growth response of the desert-adapted plant *N. sibirica* in riparian and desert habitats include flavonoid biosynthesis. Flavonoids can play a protective role in the plant’s adaptation to drought stress [12]. Usually, drought stress leads to oxidative damage, lipid peroxidation in cells, etc., which are detrimental effects to plants. Flavonoids have strong antioxidant and free-radical scavenging capabilities, which can alleviate these stress effects on plants [13]. Moreover, flavonoids can regulate water utilization and root growth of plants, improving their drought tolerance and water utilization efficiency. By promoting the exchange of substances and signals between leaves and roots, flavonoids can help plants better obtain and allocate water resources, thereby enhancing their adaptive capacity to adverse conditions [14].

This study revealed significant differentiation in the metabolic pathways of *N. sibirica* leaves between the two heterogeneous habitats (riparian and desert). In the riparian habitat, the growth adaptation strategy of *N. sibirica* mainly depends on the coordinated regulation of hormone signal transduction and lipid metabolism pathways (Table S5), a phenomenon closely associated with the soil environmental characteristics of this habitat. Based on our previous research results [9], it can be seen that the riparian habitat simultaneously possesses high soil moisture content (12.92%) and salt content (with an electrical conductivity value of 11.14 mS·cm^−1^). These dual environmental conditions jointly drive the specific regulation of the metabolic pathways of *N. sibirica*. Although high soil moisture can provide a certain water guarantee for plant growth, the salt stress caused by high salt content still exerts a significant impact on plant physiological metabolism. On the one hand, the high-salt environment tends to disrupt the osmotic balance of plant cells, leading to cell dehydration damage; the regulation of lipid metabolism pathways can adjust the composition ratio of membrane lipids, thereby maintaining the integrity and stability of cell membranes, reducing water loss and the improper penetration of salt ions. On the other hand, the hormone signal transduction pathway can synergistically regulate the plant’s stress response to salt stress: by regulating the synthesis and transmission of related hormones, it alleviates the inhibitory effect of salt stress on growth and development, enabling *N. sibirica* to adapt to the high-salt and high-humidity riparian habitat. Oxidative stress induced by the high-salt environment also damages cell membrane structures and lipid molecules, suggesting that *N. sibirica* may reduce oxidative damage by down-regulating lipid synthesis levels. This regulatory mechanism is consistent with the findings of Li et al. [15], who reported that key lipid components (e.g., phospholipids, glycolipids, fatty acids, and sphingolipids) all showed a downward trend in alfalfa after salt stress treatment.

Existing studies have shown that tryptophan, as a key precursor substance for auxin biosynthesis, its metabolic process directly affects the synthesis and accumulation of auxin, and auxin is a core hormone regulating plant growth and development [16]. The main limiting factor in the desert habitat is water shortage. This environmental stress significantly inhibits the growth and development process of *N. sibirica*, thereby causing a significant downregulation in the expression levels of metabolites related to the tryptophan metabolic pathway (Table S7). This is also the adaptive metabolic regulatory strategy of *N. sibirica* in the arid desert habitat.

Based on metabolomic data, we observed a greater number of DAMs in *N. sibirica* from desert habitats and accordingly speculated that massive metabolite accumulation contributes to drought adaptation. Nevertheless, direct physiological phenotypic evidence is lacking to establish a causal relationship between metabolite accumulation and drought tolerance. Therefore, this inference is merely proposed on the basis of molecular profiling, and further physiological and biochemical experiments will be required for subsequent validation.

### 3.3. The Transcriptional and Metabolic Responses of N. sibirica to Drought Exhibit Stage-Specificity

The KEGG enrichment results of the specific DEGs of *N. sibirica* in response to drought stress during three growth stages showed that the metabolic pathways involving genes under drought stress were the most extensive at the VE stage, covering multiple metabolic pathways such as genetic metabolism, ABC transporters, and amino acid metabolism (Figure 4). This finding indicates that the response of *N. sibirica* to drought stress at the VE stage involves multiple biological functions. Firstly, the regulation of gene expression under drought stress is associated with genetic metabolism. Under drought stress, *N. sibirica* can upregulate the expression of certain genes, which in turn enhances cell wall stability and improves the plant’s drought tolerance [17]. Secondly, ABC transporters are a class of membrane-localized carrier proteins that rely on ATP hydrolysis for energy supply and are involved in the transmembrane transport of various substances in plants, including hormones, ions, lipid secondary metabolites, and sugars. Under drought stress conditions, *N. sibirica* can adjust the expression and function of ABC transporters to balance the distribution of substances inside and outside the cells, thereby enhancing its drought resistance [18]. Moreover, the regulation of gene expression under drought stress also involves amino acid metabolism. Amino acids are not only the basic components of proteins but also key intermediate products of various metabolic pathways. Under drought stress, *N. sibirica* may enhance the synthesis pathways of some drought-resistant amino acids, such as glutamine metabolism and proline metabolism, to improve its drought resistance [19].

The KEGG enrichment results of the specific DAMs of *N. sibirica* in response to drought showed that the metabolic pathways involving metabolites under drought stress were also the most extensive at the VE stage, with significant enrichment in linoleic acid metabolism, phenylalanine metabolism, nucleotide metabolism, phenylpropanoid biosynthesis, and glycerophospholipid metabolism. Linoleic acid is an important unsaturated fatty acid. Under drought stress, plant cell membranes usually undergo changes such as unsaturated fatty acid oxidation and phospholipid molecule degradation; during this process, reactive oxygen species accumulated in the cell membrane attack unsaturated fatty acids, and linoleic acid plays a vital role in maintaining cell membrane stability [20]. Phenylalanine is an amino acid that serves as a substrate in both primary and secondary metabolic processes and is also the precursor of phenylpropanoid compounds. Under drought conditions, the phenylpropanoid biosynthesis pathway can synthesize a variety of key secondary metabolites, which are beneficial for improving the antioxidant capacity and stress resistance of *N. sibirica* [21,22]. Additionally, the supply of phenylalanine can alleviate drought stress in *N. sibirica* by regulating plant growth, photosynthesis, and antioxidant defense systems, and it has been widely used as an agronomic measure to mitigate drought stress [23]. In this study, we found that phenylalanine metabolism is involved in the drought regulation of *N. sibirica* at the molecular level, with three significantly down-regulated DAMs (phenylethylamine, phenylethylglycine, and N-acetyl-L-phenylalanine), which can enhance the antioxidant capacity of *N. sibirica* (Figure S8). Nucleotide metabolism refers to the synthesis, degradation, and regulation of nucleotides in cells, and it plays important roles in cellular signal transduction and regulation. Under drought conditions, the content of nucleotides in *N. sibirica* cells may change, participating in the regulation of a series of physiological reactions. For instance, nucleotides can regulate the opening and closing of ion channels, thereby affecting the ion balance inside and outside the cells and water homeostasis. Furthermore, nucleotides can also regulate processes such as hormone signal transduction, protein synthesis, and gene expression, helping *N. sibirica* adapt to arid environments [24]. Glycerophospholipids are the main components of cell membranes and are crucial for maintaining membrane integrity and stability [25]. Under drought conditions, five metabolites involved in the glycerophospholipid metabolic pathway in the leaves of *N. sibirica* were upregulated (Figure S8). These upregulated metabolites can promote the synthesis of glycerophospholipids rich in unsaturated fatty acids, enhance the fluidity and flexibility of cell membranes, and thus improve the drought resistance of the membranes.

### 3.4. The Role of Hormone Signal Transduction in Growth and Drought Response

Plant hormone signal transduction plays a crucial role in plant drought responses. Thus, investigating the genes involved in this pathway under drought stress is of great significance for elucidating plant adaptive mechanisms and developing drought-resistant plant varieties. Drought stress induces alterations in a series of hormone signals, among which ethylene and auxin are two key participants [26]. Through integrated transcriptome and metabolome analyses, this study found that the expression levels of AP2 family genes and ethylene response transcription factors (ERFs) in *N. sibirica* were significantly upregulated under drought stress (Figure 6). The AP2 family is an important group of transcription factors widely recognized as key regulators of stress responses. Members of this family typically contain one or more AP2 domains, which enable DNA binding and subsequent regulation of downstream gene expression [27]. Notably, ERFs, a vital subfamily of the AP2 family, can bind to stress-related promoter elements (e.g., GCC-box) to regulate the expression of drought-responsive genes, such as dehydration-protective proteins and ion regulatory proteins [28]. These results suggest that the AP2 family may exert a positive regulatory role in plant drought responses. However, it should be noted that the AP2 family varies across plant species, and different members may exhibit distinct functions and regulatory mechanisms. Therefore, future studies should focus on identifying AP2 family members in specific plant species and clarifying their regulatory mechanisms. In contrast, the expression level of auxin response factors showed an inhibitory effect under drought stress in the *N. sibirica*. Future research can utilize gene silencing and heterologous overexpression techniques to further verify the drought-resistant functions of key genes such as AP2/ERF and auxin signaling pathways in *N. sibirica*.

## 4. Materials and Methods

### 4.1. Overview of the Study Area and Source of Plant Materials

The study area is located on the north bank of Aqikesu River in the Ebinur Lake Wetland National Nature Reserve in Xinjiang, China. The plot layout is consistent with the previous study [9]. Two 100 m × 100 m plots were set perpendicular to the riverbank, named riparian habitat (RH) and desert habitat (DH), with a distance of 2400 m between them. Based on the soil moisture gradient observed in the previous study [9], RH (soil moisture content 12.92%) was used as the control habitat, while DH (soil moisture content 1.34%) was regarded as the drought-stressed habitat, with a significant difference in soil moisture content (*p* < 0.05). Subsequently, 3 30 m × 30 m plots were randomly set within each plot (plot spacing > 30 m), and 5 well-growing *N. sibirica* plants along the diagonal of each plot were selected as fixed observation objects.

### 4.2. Sample Collection

Leaves were collected using sterile knives during the three key developmental stages of *N. sibirica*. This study adopted a destructive sampling strategy, in which each individual plant was sampled only once at a single stage, to avoid the effects of repeated leaf collection on plant growth and physiological status. For each habitat, we initially selected 15 healthy and uniformly growing *N. sibirica* plants. Vegetative stage (VE, from leaf germination to bud differentiation): 5 out of the 15 plants were randomly selected for leaf collection. Flowering stage (FL, 80% flowering): 5 plants were randomly selected from the remaining 10 individuals for leaf collection. Fruiting stage (FR, 80% fruiting): The last remaining 5 plants were used for leaf collection. To minimize the effect of leaf heterogeneity, we collected the 1st to 6th leaves from the top branches of each plant, keeping leaf size as consistent as possible. The sample collection for chemical analysis, transcriptome, and metabolome was conducted in the same way during the three stages.

The collected samples were placed in: (1) envelope bags for stoichiometry analysis; (2) liquid-nitrogen tanks for transcriptome and metabolome analysis. Transcriptionome sequencing involved 18 samples (2 habitats × 3 stages × 3 biological replicates). Stoichiometry analysis and metabolome sequencing involved 36 samples (2 habitats × 3 stages × 6 biological replicates). Transcription acts as upstream molecular regulation, where transcript levels are precisely controlled by transcription factors and epigenetic marks. Intra-sample gene expression variability is limited, so three biological replicates suffice for robust detection of differentially expressed genes. Metabolites, however, arise from successive regulatory cascades of transcription and translation and exhibit far greater inter-individual metabolic heterogeneity. Accordingly, six biological replicates were initially included for metabolomic analysis in this work. All samples were analyzed in the same batch to ensure the comparability of data from each developmental stage.

### 4.3. Stoichiometry Analysis

The samples used for stoichiometry analysis were dried and ground plant samples. The organic carbon content (LOC) was determined using the potassium dichromate (K_2_Cr_2_O_7_) oxidation–external heating method. The total nitrogen content (LTN) was measured by Nessler’s colorimetric method, and the total phosphorus content (LTP) was determined by molybdenum–antimony anti-colorimetric method. Briefly, for total nitrogen determination, samples were digested with concentrated sulfuric acid and catalysts, followed by distillation and Nessler’s reagent colorimetric detection. For total phosphorus determination, samples were digested with concentrated sulfuric acid and perchloric acid and then reacted with molybdenum–antimony reagent under acidic conditions to form a blue complex, which was measured spectrophotometrically at 700 nm. All detailed analytical procedures followed the methods described in Soil Agrochemical Analysis [29].

### 4.4. Transcriptome Sequencing and Analysis

Transcriptome analysis was performed using 18 frozen leaf samples. Sequencing was conducted by Majorbio Bio-pharm Biotechnology Co., Ltd. (Shanghai, China). RNA was extracted using a TRIzol^®^ Reagent, and RNA concentration was also detected [30], followed by RNA fragmentation and reverse transcription. Individual mRNA libraries were constructed for each sample and sequenced on the Illumina NovaSeq 6000 platform.

Raw sequencing data were filtered, and sequencing error rates and GC content distributions were evaluated to generate high-quality clean reads for downstream analysis [31]. Because a reference genome is not currently available for *N. sibirica*, de novo transcriptome assembly was performed using Trinity software (v2.8.5) [32], and the assembled transcripts were used as the reference sequence. Transcripts were functionally annotated by alignment against six public databases: NR, Swiss-Prot, Pfam, COG, GO, and KEGG. Expression levels of unigenes were quantified as TPM (transcripts per kilobase per million mapped reads). A portion of the transcriptome datasets generated in this study has been deposited in the NCBI Sequence Read Archive (SRA) under accession number PRJNA1477739.

Differentially expressed genes (DEGs) were identified using thresholds of false discovery rate (FDR) < 0.05 and |log_2_fold change| > 2 [33]. Growth-related DEGs were identified from two comparisons: FL vs. VE and FR vs. FL. Drought-related DEGs were identified from three comparisons: DH_VE vs. RH_VE, DH_FL vs. RH_FL, and DH_FR vs. RH_FR.

### 4.5. Metabolomics Analysis

Metabolomic analysis was performed using 36 frozen leaf samples. Metabolite profiling was conducted by Majorbio Bio-pharm Biotechnology Co., Ltd. (Shanghai, China) using non-targeted liquid chromatography–tandem mass spectrometry (LC-MS/MS) [34]. Identified metabolites were annotated using the Human Metabolome Database (HMDB, https://hmdb.ca/metabolites, accessed on 1 September 2023) and KEGG COMPOUND Database (https://www.kegg.jp/kegg/compound/, accessed on 1 January 2023). For plant-specific secondary metabolites and plant hormones, we further verify the initial matches of HMDB using the KEGG COMPOUND Database (https://www.kegg.jp/kegg/compound/, accessed on 1 September 2023).

Differentially accumulated metabolites (DAMs) were identified based on the following criteria: variable importance in projection (VIP) > 1, *p* < 0.05, and fold change (FC) ≥ 2 or FC ≤ 0.5. Growth-related DAMs were identified from FL vs. VE and FR vs. FL, while drought-related DAMs were identified from DH_VE vs. RH_VE, DH_FL vs. RH_FL, and DH_FR vs. RH_FR.

### 4.6. Statistical Analysis

Shapiro–Wilk tests and Bartlett tests were used to assess data normality and homogeneity of variance, respectively. When normality and variance homogeneity were satisfied, two-way analysis of variance (ANOVA) was performed using the ‘aov’ function to evaluate the significance of environment (H), growth stage (S), and their interaction (H × S) on leaf stoichiometric traits of *N. sibirica*. Multiple comparisons were conducted using the least significant difference (LSD) method. For non-normally distributed data or heterogeneous variances, nonparametric Kruskal–Wallis tests were performed using the ‘kruskal.test’ function. Correlation analysis among physiological and biochemical indicators was performed using the ‘psych’ package in R.

Gene expression levels across all leaf samples were compared. Normalized gene expression data were subjected to principal component analysis (PCA) and hierarchical clustering analysis using the ‘prcomp’ function and the ‘ComplexHeatmap’ package, respectively. Permutational multivariate analysis of variance (PERMANOVA) was used to test for significant differences in gene expression among groups (habitats and stages). Venn diagrams were constructed using the ‘VennDiagram’ package to analyze the distribution of shared and unique DEGs among comparison groups.

Metabolic profiles among samples from different developmental stages were compared. Normalized metabolite data were analyzed by PCA using the ‘prcomp’ function and by orthogonal partial least-squares discriminant analysis (OPLS-DA) using the ‘ropls’ package. In univariate analysis, Student’s *t*-tests were used to calculate *p*-values, and FC was computed for each metabolite between groups.

Multi-omics integration analysis was performed to identify key genes and metabolites within critical pathways. First, two-way orthogonal partial least-squares (O2PLS) analysis was conducted for all transcriptomic and metabolomic samples to quantify the degree of association between DEGs and DAMs. Pearson correlation analysis between DEGs and DAMs was performed using the ‘psych’ package in R and visualized using Cytoscape software [35]. Then, growth- and drought-related DEGs and DAMs were separately screened based on transcriptomic and metabolomic results, and KEGG enrichment analysis was performed to identify their involved pathways. To clarify the key KEGG pathways underlying growth maintenance and drought stress responses in *N. sibirica*, cross-analysis was performed on significant pathways identified by both omics, and metabolic pathways significantly enriched in both growth and drought responses were recognized.

All statistical analyses and visualizations were performed using R software (v4.1.2).

## 5. Conclusions

This study systematically investigated the chemometric characteristics and molecular response mechanisms of *N. sibirica* under drought stress. The results revealed that habitat heterogeneity significantly altered leaf carbon and nitrogen accumulation of *N. sibirica*. The riparian habitat facilitated leaf nitrogen accumulation, whereas the desert habitat induced organic carbon enrichment to enhance stress tolerance. Furthermore, distinct growth response mechanisms of *N. sibirica* were observed between the two habitats. Plant growth in the riparian habitat was primarily modulated by hormone signal transduction and lipid metabolism pathways, while that in the desert habitat was dominated by secondary metabolite biosynthesis and amino acid metabolism. Among them, the unique metabolic pathways in the desert habitat (such as flavonoid biosynthesis and tryptophan metabolism) are potential candidate molecular bases underlying stress adaptation for its adaptation to the extremely arid environment. Differentially expressed genes and DAMs of *N. sibirica* under drought stress exhibited obvious temporal specificity, and the vegetative growth stage was identified as the critical period for drought resistance and the maintenance of fundamental life activities. Pathways including hormone signal transduction, tryptophan metabolism, alkaloid synthesis, and phenylpropanoid biosynthesis collectively formed the core regulatory network underlying the drought tolerance mechanism of *N. sibirica*. This study elucidates the molecular regulatory mechanisms by which *N. sibirica* adapts to drought stress, providing a solid theoretical foundation and valuable data support for the conservation and rational utilization of this species in desert ecosystems.

## Data Availability

The sequence data have been submitted to the NCBI Sequence Read Archive (https://www.ncbi.nlm.nih.gov/sra, accessed on 12 June 2026) under BioProject accession number PRJNA1477739.

## Supplementary Materials

The following supporting information can be downloaded at https://www.mdpi.com/article/10.3390/plants15162422/s1, Figure S1: Validation of transcriptome data by quantitative real-time PCR; Figure S2: Number of drought-responsive DEGs; Figure S3: Growth response DAM volcanic map; Figure S4: HMDB classification of 1239 core DAMs; Figure S5: The KEGG enrichment results of the unique DAMs for drought responses at different stages; Figure S6: Orthogonal partial least-squares comprehensive analysis; Figure S7: DAMs and DEGs involved in alkaloid synthesis and phenylpropane biosynthesis; Figure S8: Metabolite expression abundance bar chart; Table S1: Two-way ANOVA analysis of chemical composition characteristics; Table S2: The correlation coefficient between the chemical composition indices; Table S3: Statistics of the quality and output of the RNA-Seq libraries; Table S4: The related network properties of growth response DEGs and DAMs; Table S5: The related network properties of drought-responsive DEGs and DAMs.; Table S6: The results of the common KEGG functional enrichment analysis of growth response DAMs and DEGs; Table S7: Differentially expressed genes (DEGs) and metabolites (DAMs) involved in growth responses associated with flavonoid biosynthesis (map00941); Table S8: Differentially expressed genes (DEGs) and metabolites (DAMs) involved in growth responses associated with tryptophan metabolism (map00380); Table S9: The combined KEGG functional enrichment analysis results of drought response DAMs and DEGs; Table S10: Differentially expressed genes (DEGs) involved in drought responses associated with tryptophan metabolism (map00380); Table S11: DEGs involved in the drought response of phenylpropane biosynthesis pathways.

## Abbreviations

The following abbreviations are used in this manuscript:

DEGs	Differentially expressed genes
DAMs	Differentially accumulated metabolites
VE	Vegetative stage
FL	Flowering stage
FR	Fruiting stage
RH	Riparian habitat
DH	Desert habitat
VIP	Variable importance in projection
FC	Fold change
OPLS-DA	Orthogonal partial least-squares discriminant analysis
