# Supplementary material for: Multi-Omics Analysis of Drought and Growth Responses in Nitraria sibirica: Insights from Habitat-Specific and Developmental Comparisons
Source: Plants (Basel). 2026 Aug 8;15(16):2422. doi: 10.3390/plants15162422 (PMC13516578; doi:10.3390/plants15162422)
Supplement: Supplementary file 1 [file plants-15-02422-s001.zip › Figures S1-S8, Tables S1-S5, S7, S8, S10 and S11.pdf]

1. Supplementary figures

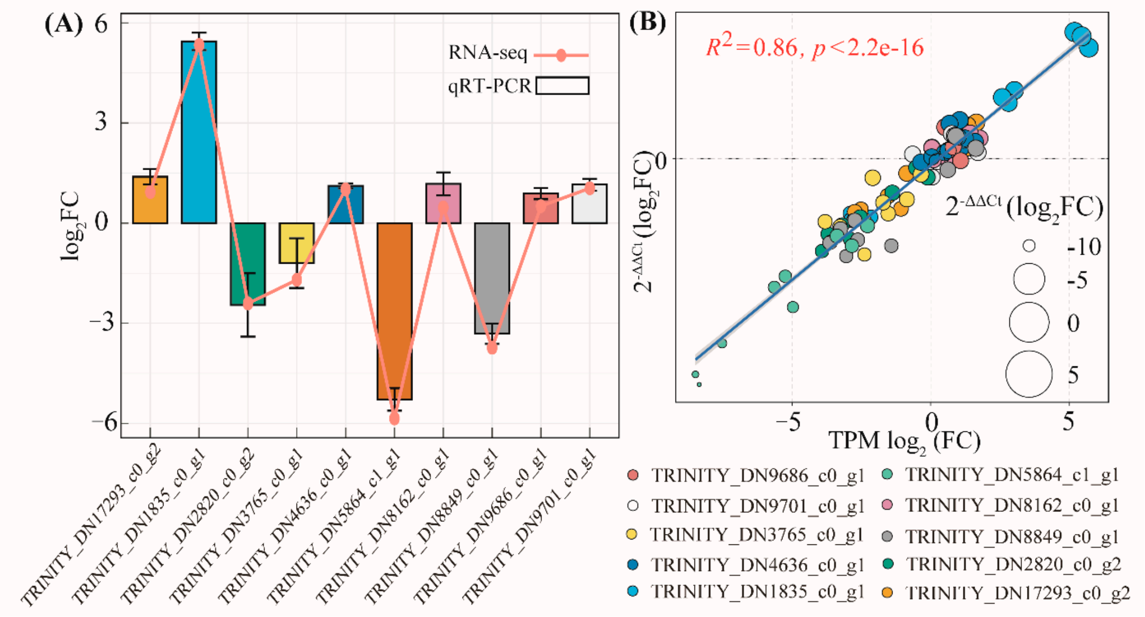

Figure S1. Validation of transcriptome data by quantitative real-time PCR. (A) The relative expression levels of 10 selected DEGs and RNA-Seq data (The broken lines represent the results of qRT-PCR, and the columns represent the results of RNA-Seq); (B) Scatter plot of correlation between gene expression levels in the transcriptome and qRT-PCR data. The PCR data and figures used in this study were from the same set of samples reported in our previous research [8].

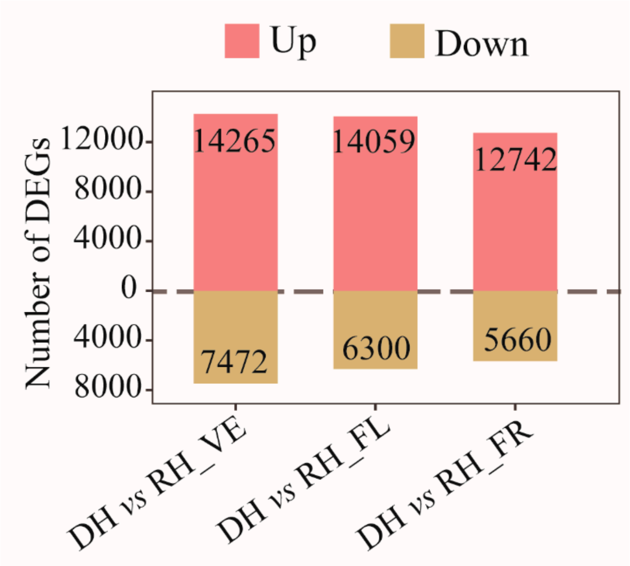

Figure S2. Number of drought-responsive DEGs. RH and DH represent riparian habitat and desert habitat, respectively; VE, FL, and FR represent vegetative, flowering and fruiting, respectively. Fig. S2 reproduced from our previous study [8]

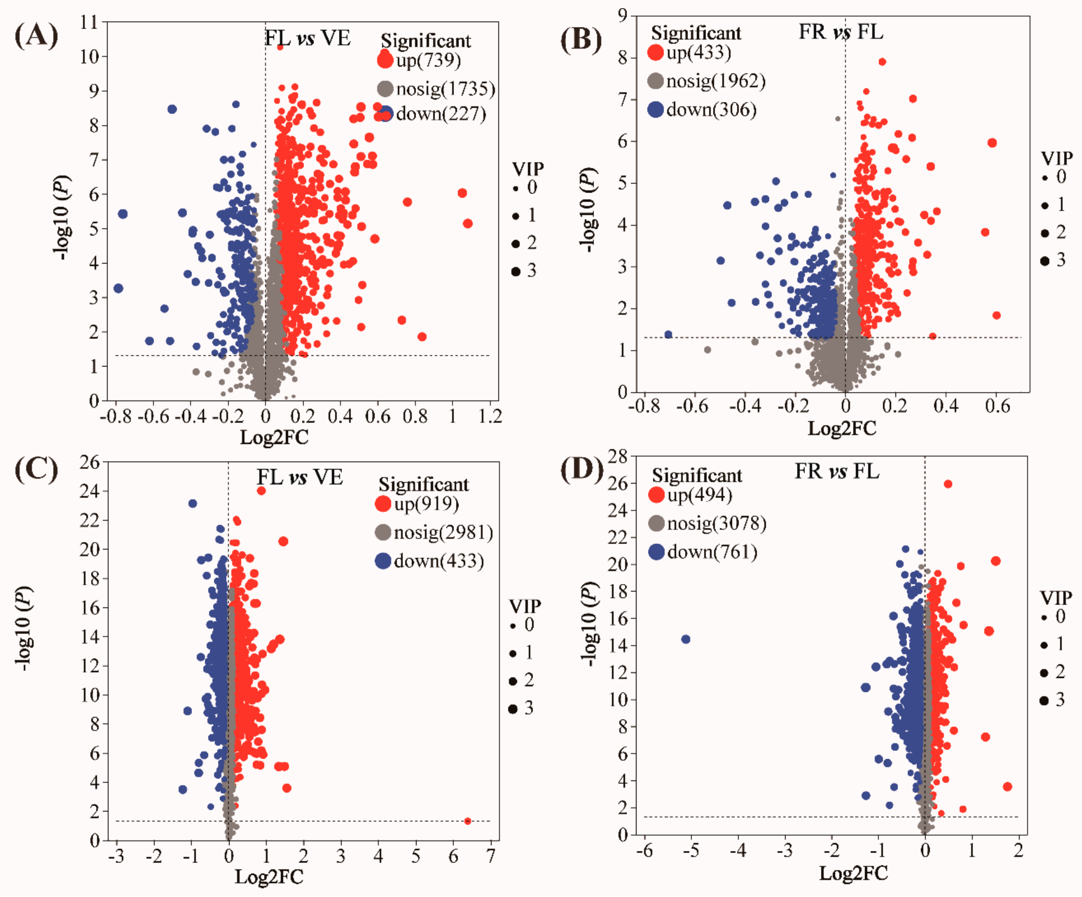

Figure S3. Growth response DAMs volcanic map. Riparian habitat comparison group (A) FL *vs* VE and (B) FR *vs* FL; Desert habitat comparison group (C) FL *vs* VE and (D) FR *vs* FL. VE, FL, and FR represent vegetative, flowering and fruiting, respectively. Each dot in the volcano map represents a specific gene, with the dot on the left representing a metabolite with downregulated expression and the dot on the right representing a metabolite with upregulated expression. Figure S3 reproduced from our previous study [9].

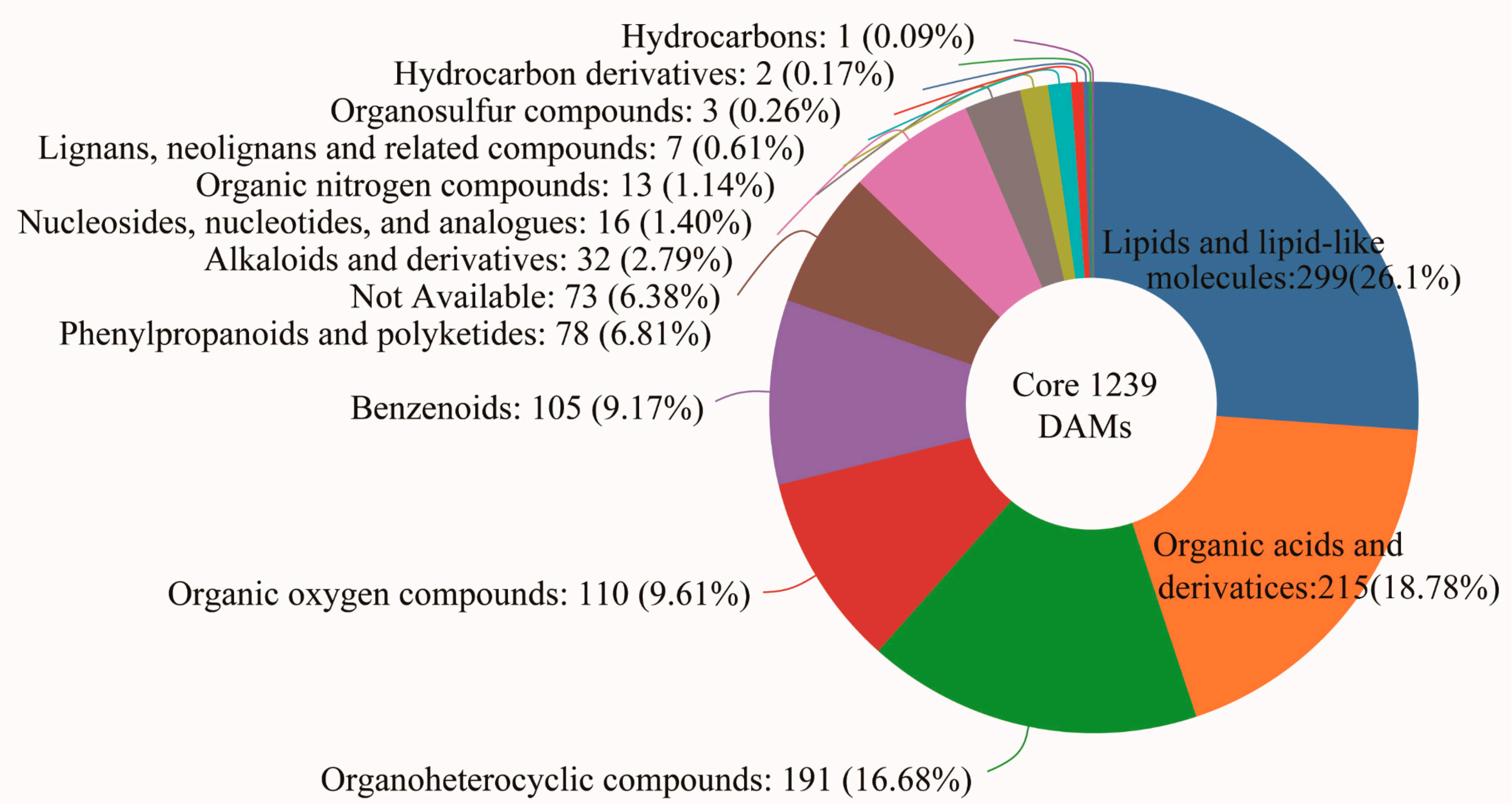

Figure S4. HMDB classification of 1239 core DAMs.

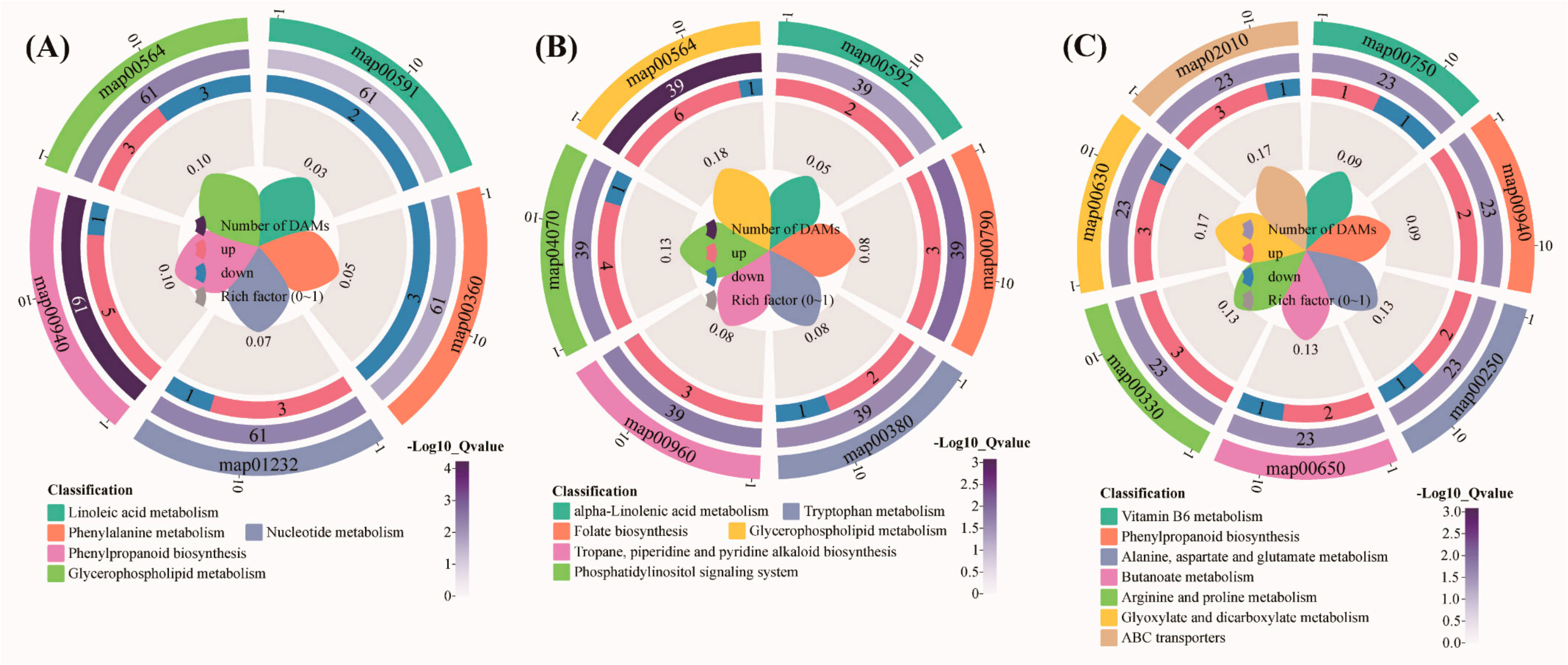

Figure S5. The KEGG enrichment results of the unique DAMs for drought responses at different stages. (A) Vegetative; (B) Flowering; (C) Fruiting.

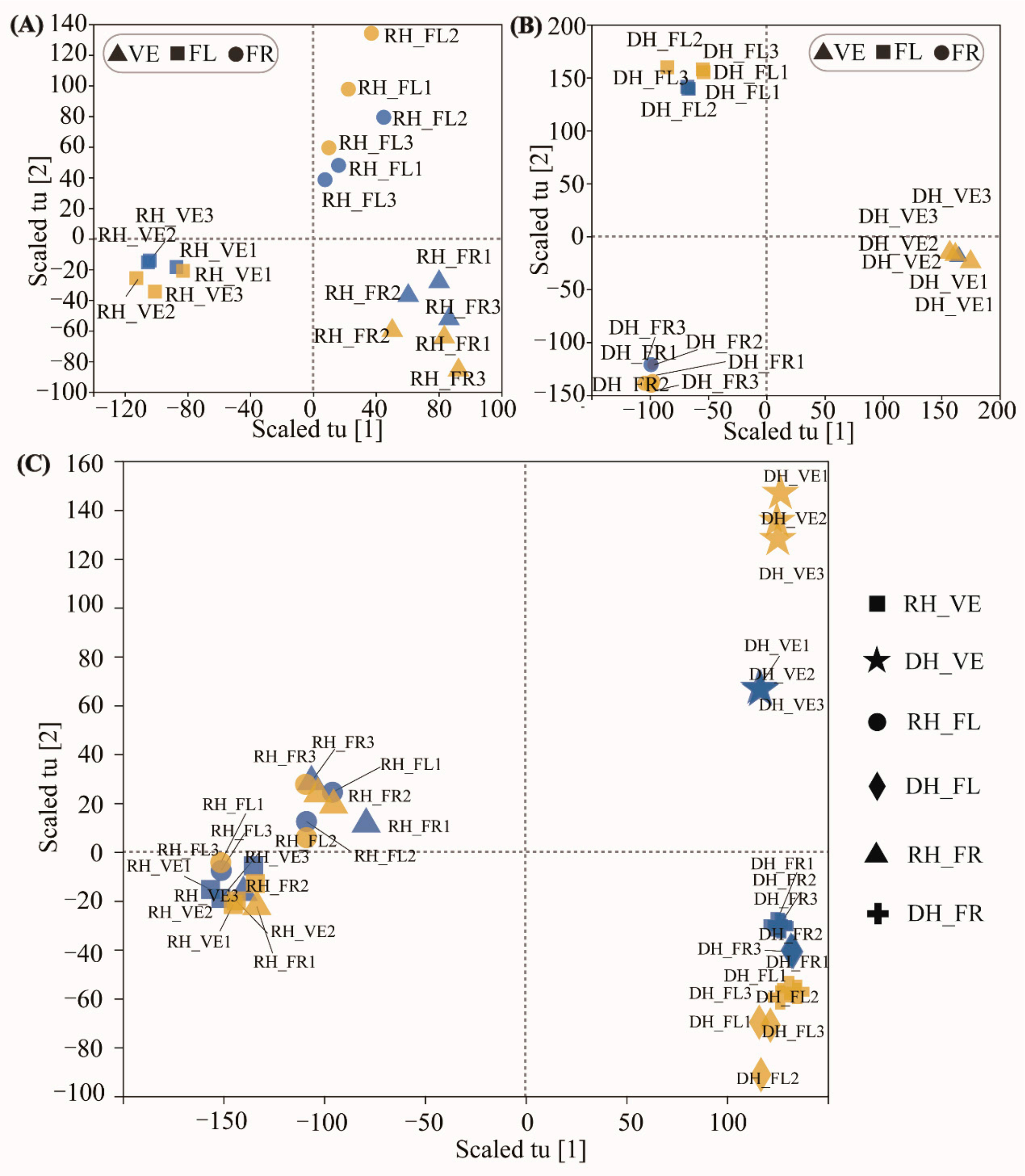

Figure S6. Orthogonal partial least squares comprehensive analysis. Growth response DEGs and DAMs in the (A) riparian and (B) desert habitats. (C) Drought response DEGs and DAMs in two habitats. RH and DH represent riparian and desert habitats, respectively. VE, vegetative stage; FL, flowering stage; FR, fruiting stage. Yellow indicates differentially expressed genes; Blue indicates differentially accumulated metabolites.

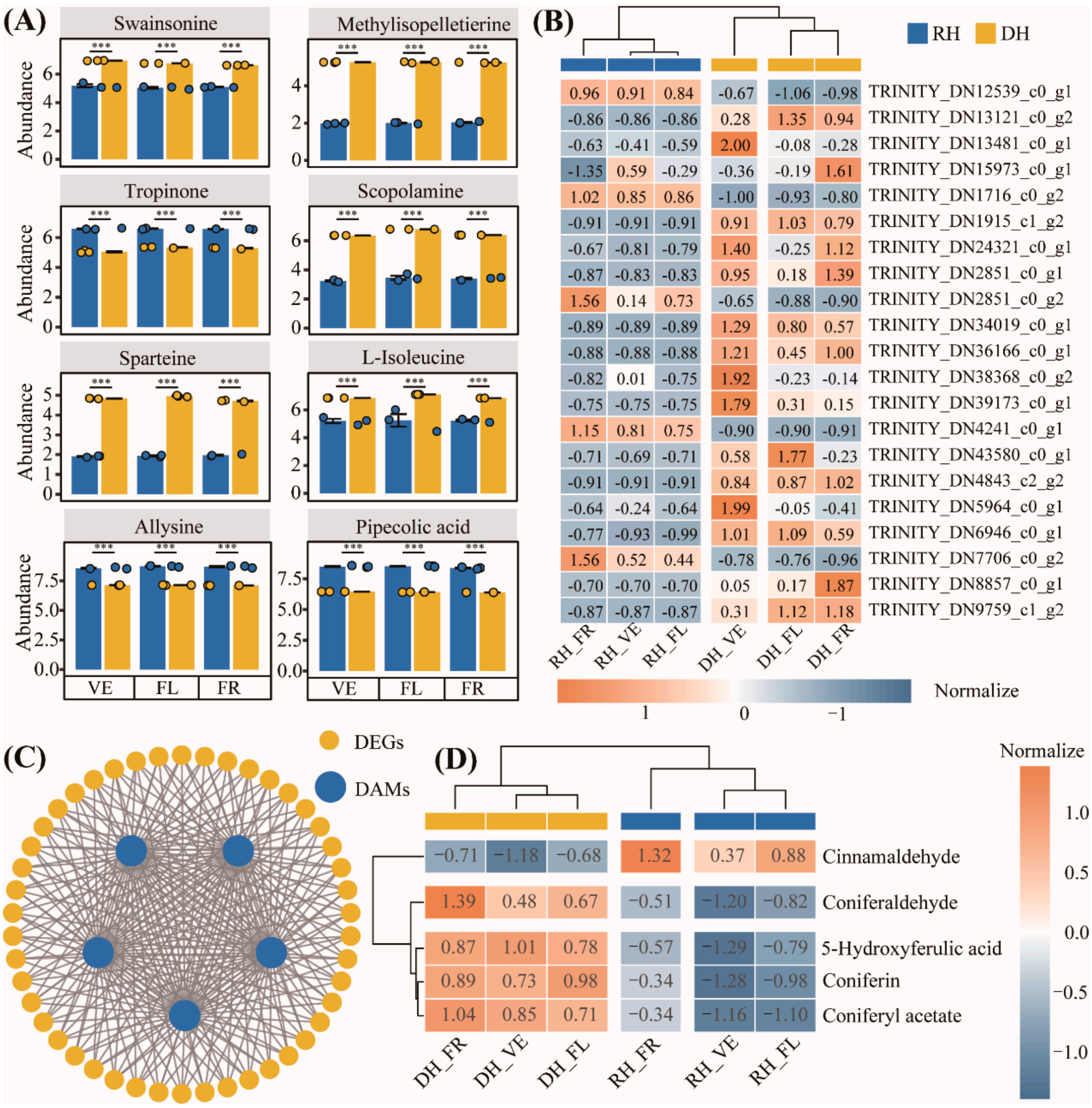

Figure S7. DAMs and DEGs involved in alkaloid synthesis and phenylpropane biosynthesis. (A) The expression abundance of DAMs in the pathway of alkaloid synthesis; (B) Cluster heat maps of DEGs in the pathway of alkaloid synthesis. (C) Maps of DAMs and DEGs networks. Yellow and blue circles represent different genes and metabolites, respectively. (D) Cluster heat maps of 5 DAMs in the pathway of phenylpropane biosynthesis. VE, FL and FR represent the vegetative, flowering and fruiting stages, respectively. RH and DH represent riparian and desert habitats, respectively.

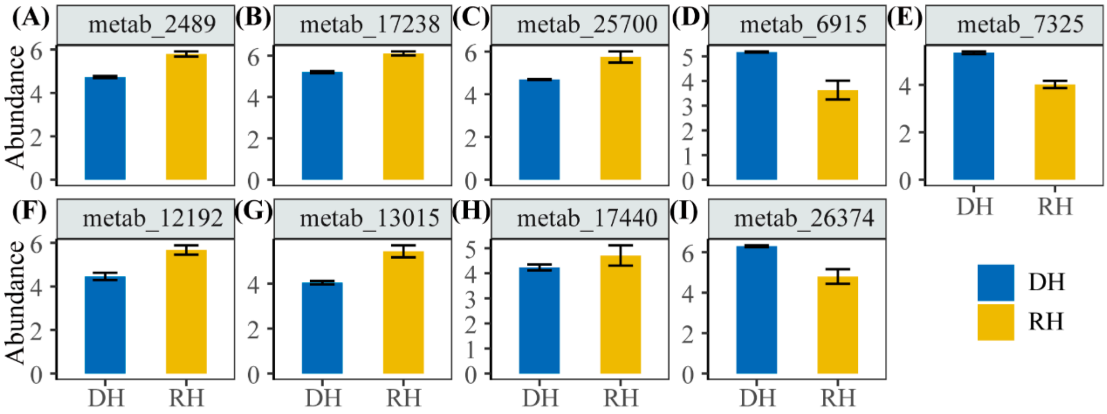

Figure S8. Metabolite expression abundance bar chart. (A) Phenethylamine. (B) Phenylacetylglycine. (C) N-Acetyl-L-phenylalanine. (D) LysoPC. (E) Phosphatidic acid (18:3/20:5). (F) Phosphatidylserine. (G) Phosphatidylglycerol phosphate. (H) Phosphatidic acid (20:5/14:1). (I) Phosphatidic acid (18:3/16:0). RH and DH represent riparian and desert habitats, respectively.

Table S1. Two-way ANOVA analysis of chemical composition characteristics.

	Plots		Stages		Stages* Plots	
	H	*P*	H	*P*	H	*P*
**C**	3.9745	0.0462	27.422	0.0000	0.8832	0.6430
**TN**	5.4822	0.0192	23.4899	0.0000	3.3692	0.1855
**TP**	0.9023	0.3422	28.1035	0.0000	3.5430	0.1701
**C:N**	6.7307	0.0095	23.3529	0.0000	3.1927	0.2026
**C:P**	2.9193	0.0875	24.0812	0.0000	6.1329	0.0466
**N:P**	4.6286	0.0314	25.2973	0.0000	3.2032	0.2016

Note: C, organic carbon; TN, total nitrogen; TP, total phosphorus; C:N, carbon to nitrogen ratio; C:P, carbon to phosphorus ratio; N:P, nitrogen to phosphorus ratio; H, test statistic value; *P*, significance probability value.

Table S2. The correlation coefficient between the chemical composition indices.

	C	TN	TP	C:N	C:P	N:P
**C**	1.00					
**TN**	−0.79***	1.00				
**TP**	−0.64***	0.08ns	1.00			
**C:N**	0.69***	−0.94***	0.04ns	1.00		
**C:P**	0.86***	−0.41*	−0.94***	0.27ns	1.00	
**N:P**	−0.67***	0.98***	−0.10ns	−0.95***	−0.23ns	1.00

Note: C, organic carbon; TN, total nitrogen; TP, total phosphorus; C:N, carbon to nitrogen ratio; C:P, carbon to phosphorus ratio; N:P, nitrogen to phosphorus ratio.*, **, and *** indicate that the F values are significant when $P \leq 0.05$, $P \leq 0.01$ and $P \leq 0.001$, respectively, and ns indicates that the F values are not significant.

Table S3. Statistics of the quality and output of the RNA-Seq libraries.

Sample	Raw reads	Raw bases	Clean reads	Clean bases (G)	Error rate (%)	Q20 (%)	Q30 (%)	GC content (%)
RH_VE1	68574914	10354812014	65867710	9645334342	0.0261	97.61	93.17	45.92
RH_VE2	53621430	8096835930	51257308	7513389400	0.0259	97.73	93.42	45.82
RH_VE3	58150592	8780739392	55694844	8204218158	0.0268	97.35	92.50	45.73
RH_FL1	71314194	10768443294	68544494	10031151306	0.0264	97.51	92.89	46.03
RH_FL2	56838858	8582667558	54592924	8028677362	0.0266	97.42	92.68	46.00
RH_FL3	55715368	8413020568	53043600	7803769570	0.0265	97.50	92.81	46.06
RH_FR1	49804854	7520532954	47991672	7111192794	0.0263	97.55	93.00	45.65
RH_FR2	46792674	7065693774	44766612	6657808418	0.0264	97.47	92.85	45.53
RH_FR3	53541428	8084755628	51571176	7655537928	0.0267	97.40	92.65	45.61
DH_VE1	43170190	6518698690	42671988	6406105081	0.0249	98.11	94.29	44.81
DH_VE2	43946504	6635922104	43605466	6536689765	0.0245	98.25	94.64	44.76
DH_VE3	40638988	6136487188	40257770	6039188096	0.0243	98.34	94.9	44.82
DH_FL1	43879290	6625772790	42595174	6405876280	0.0248	98.05	94.43	44.88
DH_FL2	42705798	6448575498	42209084	6312173547	0.0245	98.23	94.62	45.17
DH_FL3	42399418	6402312118	41921774	6282099930	0.0245	98.23	94.66	45.10
DH_FR1	42461782	6411729082	42018212	6291810287	0.0245	98.25	94.67	44.95
DH_FR2	45969462	6941388762	45604352	6832746415	0.0241	98.39	95.04	44.81
DH_FR3	49702094	7505016194	49249628	7381425559	0.0243	98.31	94.83	44.69

Note: RH and DH represent riparian and desert habitats, respectively. VE, vegetative stage; FL, flowering stage; FR, fruiting stage. The RNA-seq data used in this study were derived from the same set of samples that were reported in our previous study [8].

Table S4. The related network properties of growth response DEGs and DAMs.

Habitats	FL *vs* VE		FR *vs* FL	
	**Positive**	**Negative**	**Positive**	**Negative**
RH	111	89	72	128
DH	50	150	69	131

Note: RH and DH represent riparian and desert habitats, respectively. VE, vegetative stage; FL, flowering stage; FR, fruiting stage.

Table S5. The related network properties of drought responsive DEGs and DAMs.

Stages	DH *vs* RH	
	**Positive**	**Negative**
VE	95	105
FL	44	156
FR	73	127

Note: RH and DH represent riparian and desert habitats, respectively. VE, vegetative stage; FL, flowering stage; FR, fruiting stage.

Table S7. Differentially expressed genes (DEGs) and metabolites (DAMs) involved in growth responses associated with flavonoid biosynthesis (map00941).

Groups	ID	Names	VE	FL	FR
FL *vs* VE	metab_340	Luteolin	6.15	5.41	5.42
	metab_613	Quercetin	6.74	5.64	5.53
	metab_5744	Fustin	5.27	4.81	5.33
	metab_12292	Tricetin	5.12	3.55	4.43
	metab_12338	Phlorizine	5.64	5.05	5.42
	metab_16701	Kaempferol	5.47	4.08	4.68
	metab_18357	Naringin	2.98	5.48	2.76
	metab_19733	5-p-Coumaroylquinic acid	6.70	6.03	6.58
	TRINITY_DN74845_c0_g1	hypothetical protein JRO89_XS10G0188900 [Xanthoceras sorbifolium]	0.27	1.98	0.02
	TRINITY_DN4779_c0_g3	hypothetical protein H0E87_031531, partial [Populus deltoides]	1.79	0.29	0.30
	TRINITY_DN21071_c0_g1	licodione synthase-like [Pistacia vera]	3.16	0.27	0.08
	TRINITY_DN31116_c0_g1	hypothetical protein JRO89_XS07G0170700 [Xanthoceras sorbifolium]	0.82	11.24	2.34
	TRINITY_DN11567_c0_g1	hypothetical protein JRO89_XS05G0092500 [Xanthoceras sorbifolium]	2.38	0.34	0.00
	TRINITY_DN28110_c0_g2	flavonol synthase/flavanone 3-hydroxylase-like [Citrus sinensis]	0.15	3.61	0.20
	TRINITY_DN15389_c0_g1	leucoanthocyanidin reductase-like isoform X2 [Mangifera indica]	0.57	3.37	0.72
	TRINITY_DN2333_c0_g2	chalcone synthase [Koelreuteria paniculata]	39.03	144.34	21.06

	TRINITY_DN15118_c0_g1	hypothetical protein EZV62_018213 [Acer yangbiense]	1.56	0.33	0.72
	TRINITY_DN6729_c0_g2	hypothetical protein JRO89_XS05G0092500 [Xanthoceras sorbifolium]	33.06	64.87	58.05
	TRINITY_DN2442_c0_g1	shikimate O-hydroxycinnamoyltransferase [Citrus clementina]	74.44	22.27	27.32
FR *vs* FL	metab_2067	Dattelic acid	5.24	5.25	5.76
	metab_5168	Dihydrokaempferol	5.21	5.03	5.76
	metab_5744	Fustin	5.27	4.81	5.33
	metab_12292	Tricetin	5.12	3.55	4.43
	metab_12338	Phlorizine	5.64	5.05	5.42
	metab_16701	Kaempferol	5.47	4.08	4.68
	metab_18357	Naringin	2.98	5.48	2.76
	metab_19733	5-p-Coumaroylquinic acid	6.70	6.03	6.58
	TRINITY_DN74845_c0_g1	hypothetical protein JRO89_XS10G0188900 [Xanthoceras sorbifolium]	0.27	1.98	0.02
	TRINITY_DN3344_c0_g1	probable chalcone--flavonone isomerase 3 [Jatropha curcas]	106.25	173.14	56.77
	TRINITY_DN4779_c0_g2	flavonol synthase/flavanone 3-hydroxylase-like [Pistacia vera]	205.51	189.60	81.82
	TRINITY_DN22491_c0_g1	hypothetical protein JRO89_XS04G0216000 [Xanthoceras sorbifolium]	3.77	2.14	6.91
	TRINITY_DN31116_c0_g1	hypothetical protein JRO89_XS07G0170700 [Xanthoceras sorbifolium]	0.82	11.24	2.34
	TRINITY_DN28110_c0_g2	flavonol synthase/flavanone 3-hydroxylase-like [Citrus sinensis]	0.15	3.61	0.20
	TRINITY_DN15389_c0_g1	leucoanthocyanidin reductase-like isoform X2 [Mangifera indica]	0.57	3.37	0.72

TRINITY_DN2333_c0_g2	chalcone synthase [Koelreuteria paniculata]	39.03	144.34	21.06
TRINITY_DN13169_c0_g1	cytochrome P450 CYP73A100-like [Mangifera indica]	1.39	0.92	7.18
TRINITY_DN23551_c0_g1	Transferase domain-containing protein [Cephalotus follicularis]	5.30	5.20	1.28
TRINITY_DN256_c0_g1	PREDICTED: flavonoid 3'-monooxygenase [Theobroma cacao]	60.97	71.35	28.56

Note: VE, vegetative stage; FL, flowering stage; FR, fruiting stage.

Table S8. Differentially expressed genes (DEGs) and differentially accumulated metabolites (DAMs) involved in growth responses associated with tryptophan metabolism (map00380).

**Groups**	**ID**	**Names**	**VE**	**FL**	**FR**
FL vs VE	metab_4483	4-(2-Amino-3-hydroxyphenyl)-2,4-dioxobutanoic acid	5.37	4.79	3.91
	metab_5188	N-Methyltryptamine	4.86	4.47	4.69
	metab_10323	Indoleacetic acid	5.43	5.88	5.59
	metab_18459	L-Tryptophane	4.38	3.93	2.75
	metab_21029	Kynurenic Acid	5.78	5.39	4.84
	metab_21428	N-Acetylserotonin	4.87	4.28	4.88
	metab_22108	Xanthurenic Acid	5.13	4.61	4.11
	metab_27893	Quinoline-4,8-diol	4.46	3.92	3.09
	metab_28665	6-Hydroxymelatonin	4.73	5.43	5.35
	metab_33368	N-Methylserotonin	4.05	5.27	3.96
	TRINITY_DN25734_c1_g1	(R,S)-reticuline 7-O-methyltransferase-like [Pistacia vera])	0.11	2.80	0.13
	TRINITY_DN72185_c0_g1	Methyltransf_2 domain-containing protein/Dimerisation domain-containing protein [Cephalotus follicularis])	23.91	0.00	0.00
	TRINITY_DN29571_c0_g1	O-methyltransferase 1 [Prunus dulcis])	1.49	33.64	12.70
	TRINITY_DN21260_c1_g1	hypothetical protein JRO89_XS02G0021700 [Xanthoceras sorbifolium])	12.44	4.25	5.17

	TRINITY_DN9396_c0_g1	O-methyltransferase [Mangifera indica])	7.03	20.34	33.18
	TRINITY_DN64101_c1_g1	aldehyde dehydrogenase [Quercus suber])	0.36	0.00	3.60
	TRINITY_DN47612_c0_g1	hypothetical protein B296_00019881, partial [Ensete ventricosum])	0.06	2.64	0.75
	TRINITY_DN14055_c0_g1	(R,S)-reticuline 7-O-methyltransferase-like [Pistacia vera])	9.07	6.41	38.88
	TRINITY_DN35281_c0_g1	aldehyde dehydrogenase family 2 member B7, mitochondrial-like [Glycine soja])	0.07	1.41	0.00
	TRINITY_DN44710_c0_g1	aldehyde dehydrogenase family 2 member B7, mitochondrial-like isoform X2 [Spinacia oleracea])	0.12	1.76	0.00
	TRINITY_DN7390_c0_g1	hypothetical protein CISIN_1g010842mg [Citrus sinensis])	20.45	0.65	3.91
	TRINITY_DN303_c0_g2	hypothetical protein CUMW_191740 [Citrus unshiu])	2.32	5.40	0.76
FR vs FL	metab_537	Tryptophol	4.66	4.68	5.03
	metab_4483	4-(2-Amino-3-hydroxyphenyl)-2,4-dioxobutanoic acid	5.37	4.79	3.91
	metab_18458	Acetyl-N-formyl-5-methoxykynurenamine	5.65	5.57	4.69
	metab_18459	L-Tryptophane	4.38	3.93	2.75
	metab_19970	Formyl-5-hydroxykynurenamine	5.68	5.78	5.15
	metab_21029	Kynurenic Acid	5.78	5.39	4.84
	metab_21428	N-Acetylserotonin	4.87	4.28	4.88
	metab_22108	Xanthurenic Acid	5.13	4.61	4.11
	metab_27893	Quinoline-4,8-diol	4.46	3.92	3.09
	metab_29544	L-3-Hydroxykynurenine	5.36	5.36	5.77

metab_29953	4-(2-Aminophenyl)-2,4-dioxobutanoic acid	4.80	4.99	5.77
metab_33368	N-Methylserotonin	4.05	5.27	3.96
metab_33657	3-Methyldioxyindole	4.56	4.78	4.41
TRINITY_DN44595_c0_g1	caffeic acid 3-O-methyltransferase-like isoform X1 [Pistacia vera])	0.94	1.78	0.34
TRINITY_DN25734_c1_g1	(R,S)-reticuline 7-O-methyltransferase-like [Pistacia vera])	0.11	2.80	0.13
TRINITY_DN14581_c0_g1	hypothetical protein F0562_004963 [Nyssa sinensis])	11.68	17.54	9.28
TRINITY_DN16193_c0_g1	hypothetical protein POTOM_010838 [Populus tomentosa])	6.42	6.86	2.11
TRINITY_DN9396_c0_g2	(R,S)-reticuline 7-O-methyltransferase-like [Mangifera indica])	204.33	196.55	110.31
TRINITY_DN64101_c1_g1	aldehyde dehydrogenase [Quercus suber])	0.36	0.00	3.60
TRINITY_DN14055_c0_g1	(R,S)-reticuline 7-O-methyltransferase-like [Pistacia vera])	9.07	6.41	38.88
TRINITY_DN24934_c1_g1	UDP-glycosyltransferase 74F2 [Morella rubra])	2.39	2.64	0.41
TRINITY_DN35281_c0_g1	aldehyde dehydrogenase family 2 member B7, mitochondrial-like [Glycine soja])	0.07	1.41	0.00
TRINITY_DN17047_c0_g2	(R,S)-reticuline 7-O-methyltransferase-like [Pistacia vera])	11.42	13.89	43.62
TRINITY_DN42605_c0_g1	caffeic acid 3-O-methyltransferase-like [Mangifera indica])	0.68	1.75	0.43
TRINITY_DN13489_c0_g1	hypothetical protein JRO89_XS01G0343900 [Xanthoceras sorbifolium])	1.01	0.80	0.08
TRINITY_DN7390_c0_g1	hypothetical protein CISIN_1g010842mg [Citrus sinensis])	20.45	0.65	3.91
TRINITY_DN303_c0_g2	hypothetical protein CUMW_191740 [Citrus unshiu])	2.32	5.40	0.76

Note: VE, vegetative stage; FL, flowering stage; FR, fruiting stage.

Table S10. Differentially expressed genes (DEGs) involved in drought responses associated with tryptophan metabolism (map00380).

Gene_ID	TPM					
	RH_VE	RH_FL	RH_FR	DH_VE	DH_FL	DH_FR
TRINITY_DN14664_c0_g1	0.53	0.35	0.53	1.26	9.72	1.06
TRINITY_DN20720_c0_g2	0.00	0.00	0.00	139.21	91.28	66.86
TRINITY_DN22505_c0_g3	0.14	0.92	0.03	49.98	45.85	37.33
TRINITY_DN9072_c0_g1	0.21	0.05	0.64	79.06	123.50	54.65
TRINITY_DN6972_c0_g1	0.00	0.00	0.00	51.82	65.16	17.76
TRINITY_DN5944_c0_g1	0.00	0.00	0.00	2.33	2.32	0.22
TRINITY_DN5944_c0_g2	0.00	0.00	0.00	3.27	7.30	0.94
TRINITY_DN6011_c1_g2	0.00	0.38	0.00	1143.09	964.02	1461.43
TRINITY_DN4199_c0_g1	10.62	3.04	3.52	0.19	0.08	0.01
TRINITY_DN8157_c0_g1	0.12	0.34	0.69	7.80	16.93	5.07
TRINITY_DN15389_c0_g1	0.00	0.01	0.00	0.87	1.80	0.41
TRINITY_DN3500_c1_g1	0.02	0.01	0.01	48.33	74.75	15.41
TRINITY_DN61537_c2_g1	215.48	179.92	296.24	12.94	49.70	62.04
TRINITY_DN11624_c0_g1	1.75	8.97	0.29	0.53	0.20	0.00
TRINITY_DN35832_c0_g1	0.00	0.00	0.00	12.04	4.26	4.80
TRINITY_DN8900_c0_g1	229.90	331.14	686.91	83.20	89.81	55.61
TRINITY_DN43919_c0_g1	0.03	0.08	0.41	15.86	0.62	3.51
TRINITY_DN9453_c0_g1	88.78	36.05	77.37	13.95	9.79	15.66
TRINITY_DN9028_c0_g1	37.45	39.11	29.17	16.02	10.90	12.42
TRINITY_DN20567_c0_g1	82.40	86.65	135.86	4.17	0.00	0.00
TRINITY_DN53118_c0_g1	44.66	68.96	92.33	22.58	21.39	19.72
TRINITY_DN6634_c0_g1	0.00	0.00	0.00	62.91	50.36	37.49
TRINITY_DN9813_c0_g1	0.00	0.00	0.00	1.68	4.17	5.33

Note: RH and DH represent riparian and desert habitats, respectively. VE, vegetative stage; FL, flowering stage; FR, fruiting stage. TPM represent transcripts per kilobase per million mapped reads.

Table S11. DEGs involved in the drought response of phenylpropane biosynthesis pathways.

Gene ID	TPM					
	RH_VE	RH_FL	RH_FR	DH_VE	DH_FL	DH_FR
TRINITY_DN11009_c0_g1	3	0.21	1.23	20.44	7.53	33.06
TRINITY_DN11624_c0_g1	0.53	0.35	0.53	1.26	9.72	1.06
TRINITY_DN14162_c0_g1	1.02	0.25	0.44	5.26	4.93	3.51
TRINITY_DN14310_c0_g1	0.27	0.09	0.07	1.29	0.88	5.28
TRINITY_DN1520_c0_g1	304.32	366.22	590.61	106.3	59.57	61.93
TRINITY_DN1521_c0_g1	21.81	4.67	12.85	244.14	171.63	339.11
TRINITY_DN15389_c0_g1	0.14	0.92	0.03	49.98	45.85	37.33
TRINITY_DN15741_c0_g1	23.23	29	17.17	9.15	2.96	6.91
TRINITY_DN16208_c0_g1	54.41	60.79	89.87	9.44	7.05	8.31
TRINITY_DN18352_c0_g1	123.33	74.54	202.84	33.27	13.06	16.91
TRINITY_DN18355_c0_g1	0	0	0	10.5	7.89	20.72
TRINITY_DN18501_c1_g1	0	0	0	1.45	1.88	0.28
TRINITY_DN1862_c0_g1	14.91	9.2	9.48	74.25	33.96	90.42
TRINITY_DN20567_c0_g1	0.21	0.05	0.64	79.06	123.5	54.65
TRINITY_DN22505_c0_g3	0	0	0	2.33	2.32	0.22
TRINITY_DN22926_c0_g1	0.08	0.69	0.11	10.8	16.9	11.54
TRINITY_DN23774_c0_g1	0.27	0.98	0.24	3.69	4.09	0.91
TRINITY_DN239_c0_g1	33.04	28.47	23.54	15.77	14.53	15.44
TRINITY_DN25427_c0_g1	1.68	0.61	1.49	20.96	24.32	14.31
TRINITY_DN25427_c0_g2	0	0	0	2.69	2.65	5.97
TRINITY_DN25923_c0_g1	0	0	0	0.96	0.53	1.88
TRINITY_DN2657_c0_g1	45.16	14.04	10.5	5.95	2.54	1.33
TRINITY_DN266_c0_g1	56.86	10.37	13.64	353.35	248.55	527.71
TRINITY_DN26897_c0_g1	0.11	0.02	0.05	7.97	6.76	5.14
TRINITY_DN30718_c0_g1	0.03	0.01	0	2.48	9.93	1.9
TRINITY_DN3104_c0_g1	0.97	0	0.59	3.25	1.12	4.17
TRINITY_DN3104_c0_g2	49.62	8.78	4.24	186.95	81.17	365.49
TRINITY_DN34237_c0_g1	0	0	0	71.51	92.1	141.96
TRINITY_DN3475_c0_g2	8.5	4.11	3.21	157.79	25.36	56.65

TRINITY_DN3500_c1_g1	0	0	0	3.27	7.3	0.94
TRINITY_DN3747_c0_g2	5.77	0.3	3.23	27.07	8.74	58.82
TRINITY_DN3747_c0_g3	0	0	0	38.94	41.57	69.4
TRINITY_DN3798_c0_g1	2.52	0.23	1.12	5.94	2.3	12.41
TRINITY_DN3798_c0_g2	19.92	7.85	20.14	131.91	93.62	180.18
TRINITY_DN448_c0_g1	696.16	238.87	205.19	84.34	64.2	48.59
TRINITY_DN4542_c0_g1	3.02	0.59	1.36	45.13	37.61	14.67
TRINITY_DN53118_c0_g1	0	0.01	0	0.87	1.8	0.41
TRINITY_DN7359_c0_g1	0.51	0.96	0.49	41.44	35.16	25.91
TRINITY_DN80942_c0_g1	0.21	0	0	7.11	4.01	6.21
TRINITY_DN8664_c0_g1	60.7	29.99	32.97	925.06	720.74	1046.4
TRINITY_DN89211_c0_g1	42.63	18.72	16.09	208.31	125.66	140.75
TRINITY_DN8953_c0_g1	0	0	0	24.63	15.94	7.03
TRINITY_DN898_c2_g2	0	0	0	1.5	3.95	3.71
TRINITY_DN899_c0_g1	10.64	8.14	8.24	75.12	119.84	102.62
TRINITY_DN899_c0_g2	12.81	8.56	6.08	77.31	134.08	115.71
TRINITY_DN9453_c0_g1	0	0	0	62.91	50.36	37.49

Note: VE, FL and FR represent the vegetative, flowering and fruiting stages respectively. RH and DH represent riparian and desert habitats, respectively. TPM represent transcripts per kilobase per million mapped reads.
